# Streamline Protocol for *Arabidopsis* Apoplastic Fluid Isolation Enables a Detailed Proteomic View of the Plant Extracellular Space

**DOI:** 10.1002/pld3.70087

**Published:** 2025-07-02

**Authors:** Kuo‐En Chen, Marilee Karinshak, Richard D. Vierstra

**Affiliations:** ^1^ Department of Biology Washington University in St. Louis St. Louis Missouri USA

**Keywords:** apoplastic fluid extraction, LC–MS/MS proteomic, vacuum infiltration/centrifugation

## Abstract

The apoplastic space surrounding plant cells, encompassing the cell wall matrix, extracellular spaces, and xylem, is one of the least understood compartments within plant tissues due to its lack of limiting membranes and its unavoidable damage upon tissue homogenization. Using a streamlined vacuum‐infiltration/centrifugation protocol to enrich for the *Arabidopsis* apoplastic fluid (APF) combined with in‐depth tandem mass spectrometry, we provide an improved view of its proteome that includes over 1500 proteins possibly assigned to this compartment with minimized cytosolic contamination. Included are large and varied collections of polypeptides associated with cell wall metabolism, oxido‐reductase reactions, cell–cell signaling, proteolysis, and pathogen protection via basal defense pathways. While numerous apoplast proteins were predicted to house N‐terminal signal peptide sequences that direct extracellular secretion, many did not, suggesting widespread use of non‐classical export route(s). Among APF constituents are numerous pathogenesis‐related proteins, glycosidases, aspartyl and subtilisin‐type serine proteases, and the complement of subunits that assemble the core particle of the 26S proteasome. When this APF proteome is compared with those based on two prior isolation methods, a consensus collection of 338 polypeptides emerges that offers a comprehensive view of the core APF proteome that manages the cell wall and interfaces with the environment.

## 1 Introduction

The sequestration of metabolic and regulatory activities within cells is an essential aspect of organismal physiology and homeostasis, especially for eukaryotes that use specific compartments/locations for complicated processes ranging from DNA replication/transcription and translation to metabolism and degradation. Such compartmentalization is especially critical to plants that construct spaces devoted to photosynthesis, photorespiration, food storage, turgor, cell–cell communications, proteolysis, and biotic defense (Solymosi and Schoefs 2019; Lunn 2007). Included are chloroplasts and related plastids, distinctive microbodies/peroxisomes, protein/lipid storage bodies, embellished vacuoles, plasmodesmata, and the cellulosic cell wall that collectively enable photosynthetic energy production, concentrate food and ions, promote metabolic efficiency, and protect against reactive metabolites, deleterious side reactions, and external challenges. Consequently, full understanding of plant cells ultimately requires defining how their proteomes are selectively localized to concentrate specific functions/activities.

Whereas the organization of most plant cell compartments is reasonably well described down to their metabolic/physiological activities and protein compositions (Solymosi and Schoefs 2019; Lunn 2007), the surrounding extracellular space, commonly referred to as the apoplast, still remains poorly characterized. It occupies ~5% of the aerial tissue volume and encompasses the porous cell wall matrix, the extracellular spaces between cells, and xylem, most of which is bathed in apoplastic fluid (APF) (Rodriguez‐Celma et al. 2016; Hoson 1998). While its activities were initially thought to focus on cell wall synthesis/expansion/remodeling and nutrient acquisition, additional functions have emerged in recent years, including cell‐to‐cell and longer range communications, environmental sensing and response, wound healing, microbiome refugia, and first‐line defense against invading viral, bacterial, and fungal pathogens (Darino et al. 2022; Rodriguez‐Celma et al. 2016; Farvardin et al. 2020; Hoson 1998).

The apoplast performs these functions through a diverse collection of metabolites, enzymes, RNAs, lipids, hormones, and other signaling molecules, extracellular vesicles, and microbial byproducts, many of which are poorly characterized at present (Delaunois et al. 2013; Rodriguez‐Celma et al. 2016; Rutter and Innes 2017). It has also become increasingly clear that the composition of the APF can change during development and in response to abiotic and biotic challenges (Borniego et al. 2019; Geilfus 2017; Rodriguez‐Celma et al. 2016; Song et al. 2011; Bindschedler et al. 2008; O'Leary et al. 2016; Jiang et al. 2023). One intriguing example is the extracellular accumulation of reactive oxygen species (ROS) during pathogen attack, which are generated by a collection of apoplastic redox enzymes, including peroxidases, thioredoxins, and copper‐binding cupredoxins (Pelaez‐Vico et al. 2024). These ROS then rigidify the cell wall, induce programmed cell death for infected tissues, and activate defense pathways as part of a pathogen‐triggered immune response (PTI) (Bolwell et al. 2002; Mata‐Perez and Spoel 2019; Farvardin et al. 2020; Roussin‐Leveillee et al. 2024). Also related is the apoplastic deposition of tiny/short, circular, and long‐noncoding RNAs complexed with RNA‐binding proteins either in free or vesicle‐encapsulated forms for microbial defense (Baldrich et al. 2019; Cai et al. 2018; Zand Karimi et al. 2022). Clearly, describing the composition, functions, and dynamics of the APF has become increasingly important.

Main challenges in defining the apoplast are (i) the lack of boundaries as it encompasses the diffusional space external to the plasma membrane, (ii) the tangled mesh of cell wall polymers that hinder extraction, (iii) its low protein concentrations as compared to intracellular compartments, and (iv) its unavoidable loss of integrity upon tissue homogenization. These concerns necessitate methods to extract large volumes of APF, which is typically limiting, in a way that will release the trapped constituents while minimizing tissue/cell breakage. The approach most commonly employed to avoid tissue damage is to infiltrate plant tissues with aqueous solutions under a weak vacuum and gently collect the introduced fluid by low *g*‐force centrifugation. Various iterations of this vacuum infiltration/centrifugation (VIC) strategy have been applied to a number of species, including *Arabidopsis* (Huang et al. 2021; Boudart et al. 2005; Jiang et al. 2023; Borniego et al. 2019; Rutter et al. 2017), rice (Song et al. 2011), maize (Witzel et al. 2011), sorghum (Chaya et al. 2024), tobacco (Grosse‐Holz et al. 2018), beets (Ceballos‐Laita et al. 2015), and grape (Delaunois et al. 2013), typically using leaves as the tissue source. Unfortunately, many of the APF preparations appear contaminated with presumed intracellular proteins, especially those from chloroplasts, implying that improved extraction methods are needed. Moreover, the resulting preparations were often examined by two‐dimensional electrophoresis and/or low‐resolution mass spectrometry (MS), indicating that deeper catalogs are possible using more sensitive MS approaches.

Here, we attempted to better define the APF by simultaneously testing a streamlined VIC approach recently developed for *Arabidopsis* leaves (Zand Karimi et al. 2025) against two recent isolation methods (Rutter et al. 2017; Huang et al. 2021), using various biochemical assays and in‐depth proteomic comparisons by high‐resolution liquid chromatography coupled to tandem mass spectrometry (LC–MS/MS). Collectively, the LC–MS/MS data substantially increased the repertoire of proteins likely found in the apoplast, thus expanding functional appreciation of this compartment. A consensus APF proteome dataset of 338 proteins was also generated by aggregating the protein lists identified from the three APF isolation methods; this core catalog presumably represents abundant apoplastic constituents whose functions highlight the hydrolytic capacity of this compartment and its roles in pathogen defense.

## 2 Materials and Methods

### 2.1 Plant Materials and APF Isolation

Plants from the *Arabidopsis thaliana* ecotype Col‐0 were cultivated in a growth chamber under a short‐day photoperiod (8‐h light/16‐h dark) at 22°C for 45–50 days. For all methods, APF obtained by VIC used deionized water containing 50‐μM ATP (pH ~6.0) as the infiltrate. For the total lysate, the tissue was pulverized at liquid nitrogen temperatures with a mortar and pestle, homogenized in 50‐mM HEPES (pH 7.5), 5‐mM Na_2_EDTA, and 2‐mM DTT, and clarified by centrifugation at 12,000 × *g*. For the APF samples, the fluid was frozen, freeze‐dried under a vacuum, and stored at −20°C for subsequent MS analysis, SDS‐PAGE, and immunoblot assays.

Method 1 was based on the protocol previously outlined by Rutter et al. (2017), which was then described in greater detail (Rutter et al. 2017). For this study (Figure S1A), whole undamaged rosettes were harvested by cutting the plants at the root/shoot junction and rinsing the rosettes three times with deionized water to remove soil particles. The rosettes were then submerged and infiltrated for 20 s under a mild vacuum generated by hand with a French press. Excess solution was removed by gently shaking and then blotting the rosettes dry with paper towels. The dried tissue was loaded into a 60‐mL syringe with the shoot ends oriented downward, and the syringe was secured into a larger conical tube. The setup was centrifuged at 700 × *g* for 20 min at 4°C (Rutter et al. 2017); the extracted fluid was collected and clarified through a 0.2‐μm Acrodisc syringe filter (Pall Corp.) to generate the APF.

APF isolation Method 2 was based on the protocol described by Huang et al. (2021) with minor modifications (Figure S1B). Fully expanded and undamaged leaves from ~35 plants were dissected from the rosettes by cutting at the rosette/petiole junction, and washed three times with deionized water. Groups of 10 leaves were infiltrated in batches using a 200‐mL syringe under a hand plunger‐generated mild vacuum for 20 s followed by a slow release. Excess buffer was removed by blotting with paper towels. The petioles were excised from the leaves at the leaf/petiole junction with a razor blade; the leaves were then bundled with surgical tape onto small plastic sticks and positioned inside 60‐mL conical tubes with the tip of each leaf oriented upward. These nested tubes were centrifuged at 900 × *g* for 10 min at 4°C; the internal fluid was then filtered through a 0.2‐μm Acrodisc syringe filter to generate the APF. To simplify the method described by Huang et al. (2021), we omitted the two additional centrifugation steps used for further clarification (i.e., centrifugation at 2000 × *g* followed by 10,000 × *g*).

The streamlined method detailed here (Method 3) was adapted from the protocol described by Zand Karimi et al. (2025) (Figure S1C). Fully expanded and undamaged leaves from ~35 plants were excised at the rosette/petiole junction, washed three times with deionized water, and blotted dry with paper towels. Groups of 10 leaves were infiltrated in batches using a 60‐mL syringe filled with 40–50 mL of 50‐μM ATP sufficient to cover the leaves. A mild vacuum was generated for 20 s with a hand plunger followed by a slow release. A second infiltration was performed if needed to ensure complete filtration. The 10 leaves were then blotted dry and suspended individually in a 60‐mL syringe with the petioles oriented up. The internal fluid was collected by centrifugation at 600 × *g* for 20 min at 4°C into a nested conical tube; the fluid was then clarified through a 0.2‐μm Acrodisc syringe filter to generate the APF.

### 2.2 SDS‐PAGE and Immunoblotting

For SDS‐PAGE and immunoblot analyses, a defined quantity of total lysate and APF protein samples was mixed with 3 μL of 4× SDS sample buffer (50‐mM Tris–HCl [pH 7.5], 100‐mM DTT, 2% SDS, 0.1% bromophenol blue, 10% glycerol), incubated at 80°C for 10 min, and subsequently placed on ice for 5 min. Following SDS‐PAGE, proteins were detected in the gels by staining with Coomassie blue.

For immunoblot analysis, proteins were electrophoretically transferred onto 0.2‐μm PVDF membranes (Amersham). Membranes were blocked at room temperature for 1 h with 10% (w/v) non‐fat milk in PBST buffer (137‐mM NaCl, 2.7‐mM KCl, 8‐mM Na_2_HPO_4_, 2‐mM KH_2_PO_4_, and 0.1% [v/v] Tween‐20), followed by a 1‐h incubation with primary antibodies diluted in PBST. Proteins of interest were detected using primary rabbit antibodies at a 1:5000 dilution, including RBCL (BIOSS, bs6988R), cFBPase (Agrisera, AS04043), actin (Agrisera, AS132640), histone H3 (Agrisera, AS10710), catalase1 (PhytoAB, PHY3681S), RPS6 (PhytoAB, PHY7297S), and RPL13‐1 (Agrisera, AS132650). Rabbit antisera prepared against various proteasome subunits were as described (Book et al. 2010) and were used at a 1:5000 dilution. After incubation with primary antibodies, membranes were washed three times with PBST (10 min for each wash) for a total of 30 min followed by incubation for 1 h with the secondary antibody, anti‐rabbit IgG conjugated to horseradish peroxidase (HRP; Agrisera, AS09602; 1:5000), diluted in PBST. The membranes were then washed three times with PBST for a total of 45 min. HRP‐mediated chemiluminescence was detected using the SuperSignal West Pico PLUS chemiluminescent substrate (Thermo Scientific); the images were captured with a CCD camera. Chloroplast contamination was assayed by extracting the samples in 80% ethanol followed by spectrophotometric quantification of chlorophyll at 600 nm.

### 2.3 Protein Sample Preparation and Mass Spectrometry

LC–MS/MS were conducted as previously described (Zand Karimi et al. 2025; McLoughlin et al. 2018) using the APF and total lysate extracted from leaves from the same developmentally aged plants. The lyophilized APF and total lysate samples were dissolved in 50 μL of 8‐M urea and measured for protein concentration using the Pierce BCA Protein Assay Kit (Thermo Scientific). For trypsin digestion, 80 μg of protein from each sample was first reduced at room temperature for 1 h in 10‐mM dithiothreitol (DTT), followed by alkylation with 20‐mM iodoacetamide (IAA) for 1 h in the dark. The reactions were quenched with 20‐mM DTT, and the samples were diluted with 900 μL of 25‐mM ammonium bicarbonate to reduce the urea concentration to below 1 M. Digestions were performed overnight at 37°C with sequencing‐grade modified porcine trypsin (Promega) at a trypsin‐to‐protein ratio of 1:50. The resulting peptides were lyophilized to a volume lower than 50 μL, acidified with 10% trifluoroacetic acid until the pH was below 3.0, and desalted and concentrated using Pierce C18 Tips (Thermo Scientific) following the manufacturer's instructions. Peptides were eluted in 50 μL of 75% acetonitrile and 0.1% acetic acid, lyophilized again, and resuspended in 15 μL of 5% acetonitrile and 0.1% formic acid for LC–MS/MS analysis.

Nano‐scale LC separation of the tryptic peptides was performed using a Dionex Ultimate 3000 Rapid Separation system equipped with a 75 μm × 25 cm Acclaim PepMap RSLC C18 column (Thermo Fischer Scientific) in combination with a 2‐h linear 4%–36% acetonitrile gradient in 0.1% formic acid and a flow rate of 250 nL/min (McLoughlin et al. 2018; Zand Karimi et al. 2025). Eluted peptides were analyzed online by a Q‐Exactive Plus spectrometer (Thermo Fisher Scientific) in the positive electrospray ionization (ESI) mode. Data‐dependent acquisition of full MS scans (mass range of 380–1500 m/z) at a resolution of 70,000 was collected, with the automatic gain control target set to 3 × 10^6^ and the maximum fill time set to 200 ms. High‐energy collision‐induced dissociation fragmentation of the 15 strongest peaks was performed with an intensity threshold of 4 × 10^4^ counts and an isolation window of 3.0 *m*/*z* and excluded precursors that had unassigned, +1, +7, +8, or > +8 charge states. Tandem MS scans were conducted at a resolution of 17,500, with an automatic gain control target of 2 × 10^5^ and a maximum fill time of 100 ms. Dynamic exclusion was performed with a repeat count of 2 and an exclusion duration of 30 s, while the minimum MS ion count for triggering tandem MS was set to 4 × 10^3^ counts. Peptides obtained from each method were identified by LC–MS/MS performed sequentially, each with three biological replicates analyzed by two technical replicates. The MS runs were performed as a batch in alternating sequence among the samples (i.e., Method 1—sample 1, Method 2—sample 1, Method 3—sample 1, Method 1—sample 2 and so on) to minimize technical variations within the collection.

### 2.4 Proteomics Data Analysis and Processing

The MS/MS datasets were queried by Proteome Discoverer (version 2.5; Thermo Fisher Scientific) against the *Arabidopsis thaliana* Col‐0 proteome database (Araport11_pep_20220914), downloaded from TAIR (http://www.tair.com/). Pairs of raw MS2 files were analyzed as fractions, resulting in two technical replicates per sample. Peptides were assigned by SEQUEST HT, allowing a maximum of two missed tryptic cleavages, a minimum peptide length of 6, a precursor mass tolerance of 10 ppm, and fragment mass tolerances of 0.02 Da. Carbamidomethylation of cysteines and oxidation of methionines were specified as static and dynamic modifications, respectively. False discovery rates of 0.01 (high confidence) and 0.05 (medium confidence) were used to validate peptide spectral matches. Label‐free quantifications based on MS1 precursor ion intensity were performed in Proteome Discoverer with a minimum Quan value threshold set to 0.0001 for unique peptides; the “3 Top N” peptides were used for area calculation. Processed proteomic data generated using Proteome Discoverer 2.5 are provided in Dataset S1.

The proteomic data were normalized based on the total MS1 ion intensity of each run to equalize values across all injections (McLoughlin et al. 2018). For each biological replicate, the two technical replicates were averaged; if the protein was detected in only one technical replicate, its value was used as is. Proteins were considered for further analysis only if it was detected in all three biological replicates within a single dataset, to ensure high reliability. For volcano plots, the data were Log_2_‐transformed and statistically analyzed using Perseus (v2.1.3.0), with visualization performed in GraphPad Prism (version 10). Statistic differences based on three biological replicates were determined using Student's *t*‐test (Log_2_ FC ≥ 1 or ≤ −1, *p* < 0.05).

### 2.5 Bioinformatic Analysis

PCA plots were generated by Perseus (v2.1.3.0). GO analyses were conducted using the g:GOSt algorithm within the g:Profiler platform (v3.10.178) (http:://biit.cs.ut.ee; Kolberg et al. 2023). Protein locations were based on the GO classifications (https://biit.cs.ut.ee/gprofiler/gost) and by an improved apoplast protein list described by Zand Karimi et al. (2025). GO‐annotation categories shown here were selected based on their uniqueness, *p*‐values of significance, and degrees of completeness. Signal and transit peptide sequences were predicted using TargetP2.0 (https://services.healthtech.dtu.dk/services/TargetP‐2.0/) (Almagro Armenteros et al. 2019). The prediction for apoplast‐targeted proteins specifically employed ApoplastP (https://apoplastp.csiro.au/) (Sperschneider et al. 2018). The defense‐related proteins marked in Table 1 are based on the information for Gene Ontology Biological Processes in BioGRID^4.4^ (https://thebiogrid.org/). NN scores in Table S1 were generated in SecretomeP 2‐0 (https://services.healthtech.dtu.dk/services/SecretomeP‐2.0/).

## 3 Results

### 3.1 Comparison of Methods Used to Isolate the *Arabidopsis* APF

For an improved look at the *Arabidopsis* APF, we simultaneously examined by biochemical assays and in‐depth proteomics samples generated by our streamlined VIC protocol as compared to those obtained using two recently published methods (Huang et al. 2021; Rutter et al. 2017). The source tissues were from stage‐2, non‐infected *Arabidopsis* rosettes grown for 45–50 days under a short‐day photoperiod in an environmental chamber just prior to bolting and well before emergence of senescence symptoms (Borniego et al. 2019). Previous studies suggested that apoplast extraction was best using water alone as the infiltrate (Witzel et al. 2011; Buscaill et al. 2019); here, we added 50‐μM ATP (pH ~ 6.0) given its known presence in the plant APF (~1–5 μM; Song et al. 2006) and its need for maintaining proteasome stability (Book et al. 2010), whose proteomics was one emphasis of this study. As recommended (Lohaus et al. 2001), we also lowered the centrifugal *g* force (600 × *g*) for collecting the APF to better minimize tissue/cell damage.

Method 1 from Rutter et al. (2017) used the entire rosette as described, which was separated from the roots, washed, vacuum infiltrated with a French press, blotted dry, and extracted for APF by centrifugation of the rosettes in random orientations at 700 × *g* for 20 min followed by clarification of the fluid using a 0.2‐μm filter (Figure S1A). Method 2 from Huang et al. (2021) used only leaves, which were dissected at the petiole/stem junction, washed, blotted dry, and vacuum infiltrated with a hand‐held plunger. After removing the petioles, the leaves were bundled and centrifuged at 900 × *g* for 10 min with the leaf‐base cut sites pointing down as described. For the published method (Huang et al. 2021), this fluid was then clarified by several centrifugation and filtration steps to finally generate the APF. Here, we simplified APF clarification to just a single filtration step after centrifugal collection (Figure S1B).

For our improved VIC method (Method 3), we used leaves that were dissected at the petiole/stem junction, washed, slowly vacuum infiltrated with a hand‐held plunger for 1–2 cycles to ensure that all leaves were infiltrated, and blotted dry (Figure S1C). The dried leaves in lots of 10 were individually hung and centrifuged without bundling at 600 × *g* with the cut petioles pointing up. Importantly, this orientation forced APF exit primarily through the stomata and hydathodes, and not the petiole cut ends, thereby minimizing contamination from phloem and fluid exuding from the dissection sites. This fluid was then subjected to a single clarification step using a 0.2‐μm filter. For all three methods, the APF was frozen immediately to avoid post‐extraction proteolysis. Further analyses of particulate material, large complexes, and extracellular vesicles were possible for all three methods by pelleting the material at 100,000 × *g* (Cai et al. 2018; Rutter et al. 2017; Zand Karimi et al. 2025). To minimize sampling bias among the three protocols both in sample preparation and MS analysis, the plants were grown simultaneously, the biological replicates of leaf APFs were collected in randomized sequence, and the MS data were obtained as a batch with the biological and technical replicates from the three protocols analyzed in alternating sequence.

### 3.2 Biochemical Comparisons of APF Extraction Methods

**FIGURE 1:**
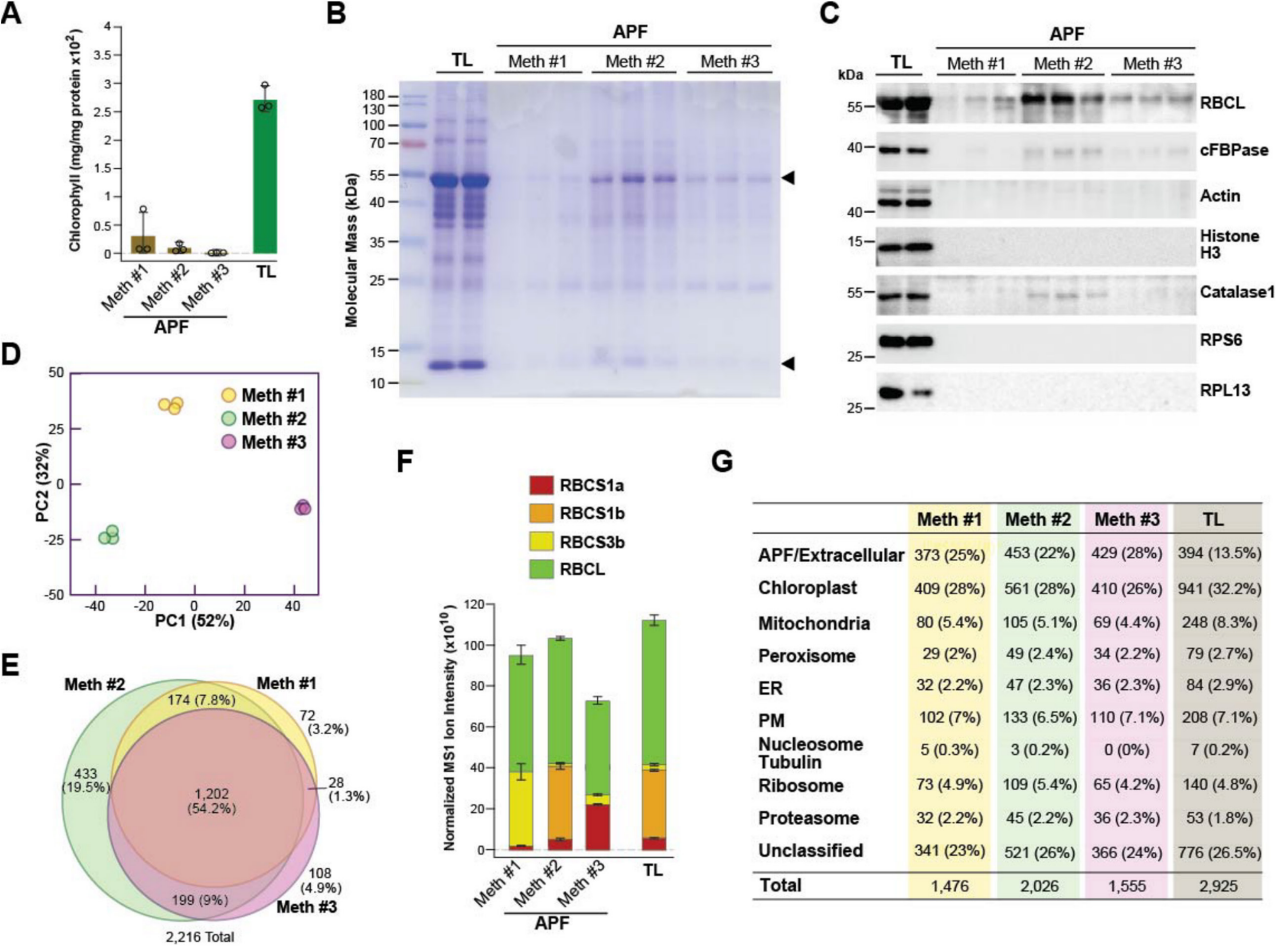
Biochemical comparisons of the streamlined VIC method (Method 3) used to isolate the APF from *Arabidopsis* leaves (Zand Karimi et al. 2025) with two prior APF isolation methods (Method 1, Rutter et al. 2017, and Method 2, Huang et al. 2021). Each sample was prepared as schematically illustrated in Figure S1. (A) Chlorophyll contamination in APF isolated by the three methods as compared to that present in total leaf lysates (TL). Each bar represents the average (±SD) of three biological replicates. Individual data points are included. (B) SDS‐PAGE and Coomassie blue staining of total protein from the APF preparations as compared to that from the TL. Arrowheads indicate the Rubisco large and small subunits. Shown are 10 μg of protein from two and three biological replicates of the TL and APF, respectively. (C) Immunoblot analyses of the APF and TL samples from panel (B) with antibodies against proteins from various intracellular compartments/protein complexes. RBCL, Rubisco large subunit (chloroplast). cFPBase, cytosolic fructose‐1,6‐bisphosphatase (cytoplasm). Actin (cytoplasm). Histone H3 (nucleus). Catalase‐1 (peroxisome). RPS6 and RPL13 proteins from the ribosome small and large subunits respectively (ribosome). (D–G) Proteomic comparisons of trypsinized samples obtained from the three methods by LC–MS/MS. Protein identification required at least one unique peptide detected in at least one of the two technical replicates for each of the three biological replicates. Data for all Rubisco large and small subunits detected were included in the analysis. (D) PCA of the samples obtained by the three VIC methods. The values were determined from log_2_‐transformed MS1 precursor ion intensities (*n* = 3 biologically independent samples). (E) Venn diagram showing the numbers and percentages of overlapping and unique proteins cataloged by MS/MS using the three isolation methods. (F) MS/MS quantification of Rubisco contamination in the apoplast derived from the three isolation methods as compared to data for the TL. Levels of chloroplast‐encoded large subunit (RBSL; AtCG00490) and nuclear‐encoded small subunit isoforms (RBCS1a, RBSC1b, and RBCS3a) were quantified based on the MS1 precursor ion intensities (*n* = 3 biologically replicates ± SD). (G) Compartment/protein complex distributions of proteins identified by LC–MS/MS after APF isolation by the three methods as compared to those of the TL. Shown are the total numbers and percentages of total proteins assigned to each location by GO. Unclassified represent proteins not yet assigned to a compartment/complex by GO. ER, endoplasmic reticulum. PM, plasma membrane. LC–MS/MS data for three biological replicates of the TL obtained from leaves grown and processed under identical conditions were from Zand Karimi et al. (2025).

To assess the extent of intracellular contamination using our improved VIC strategy (Method 3) versus Methods 1 and 2, we assayed the levels of several cytosolic markers, including chlorophyll and Rubisco large and small subunits (chloroplasts), cytosolic fructose‐1,6‐bisphosphatase (cFBPase), actin, and reference ribosomal proteins from the large and small subunit complexes—RPS6 and RPL13, respectively (cytoplasm), catalase‐1 (peroxisomes), and histone H3 (nucleus) by spectrophotometric measurements, SDS‐PAGE, or by immunoblotting the extracts. As shown in Figure 1A–C, Method 2, which extracted APF from leaf tips oriented up, acquired the most intracellular contamination, especially for Rubisco, cFBPase, and catalase, while Methods 1 and 3 had noticeably less, especially for chlorophyll and Rubisco, suggesting that centrifugal extraction of the fluid after pointing the cut leaf ends down compromised APF purity.

For further analysis, we subjected the biological replicates prepared by the three VIC methods to LC–MS/MS, with each sample analyzed by two technical replicates, using a prior dataset examining the total leaf lysate (TL) grown and analyzed under identical conditions as a reference (Zand Karimi et al. 2025). In total, 1476, 2026, and 1555 APF proteins were detected by the three methods, respectively, which surprisingly separated into tight clusters by principal component analysis (PCA) based on protein abundance calculated from the MS1 scans (Figure 1D), indicating that the protein profiles generated by the three were statistically distinct despite using relatively similar tissues and VIC protocols. Comparisons of all three protein catalogs revealed a common set of 1202 APF proteins, with Method 2 having the largest number of unique members (433 versus 72 and 108, respectively) (Figure 1E). From quantification of Rubisco large and small subunit levels based on the cumulative MS1 precursor ion intensities of all corresponding peptides, we found that Method 3 displayed the least contamination from this chloroplast protein complex, with less found for both the chloroplast‐encoded large subunit (RBCL) and the combined accumulation of the three isoforms for the nuclear‐encoded small subunit (RBSC1a, RBSC1b, and RBCS3b) (Figure 1F). Interestingly, Method 3 accumulated higher levels of RBSC3b as compared to the two other methods, the reason(s) for which we do not yet understand (see below).

We then compared the cellular locations for the proteins in each MS/MS dataset as compared to the 2925 proteins identified in the total leaf lysate, using protein numbers as a measure based on peptide spectral matches assigned to each compartment/complex (regardless of abundance). The protein location assignments used were mainly derived from the *Arabidopsis* Information Resource (TAIR; https://www.arabidopsis.org). The exception was the apoplast/extracellular list; here, we aggregated previous proteomic datasets from samples enriched for either the apoplast, extracellular spaces, or the cell wall (see Zand Karimi et al. 2025). In line with lower levels of cytosolic contamination seen biochemically, Method 3 had the highest percentage of predicted APF proteins measured by protein numbers (28%) and the lowest percentages of intracellular proteins such as those assigned to chloroplasts, mitochondria, nucleosomes/tubulin, and ribosomes (Figure 1G). Collectively, all the analyses point to our streamlined VIC method as isolating the APF with the least contamination.

For further assessments of contamination, we reanalyzed the proteomic data based not on detected protein numbers for each compartment/complex, but by the collective protein abundances for each, as quantified by combined MS1 precursor ion intensities. This quantitative approach accommodates the fact that such VIC strategies will not likely create “clean” apoplast samples, together with the understanding that the apoplast, with its low protein concentration (Rodriguez‐Celma et al. 2016), can be easily overwhelmed by protein‐rich intracellular compartments regardless of the enrichment strength. For example, the Rubisco complex alone can rise to 50% of total protein in leaves and often far exceeds (based on MS1 intensity) the estimated levels of all other *Arabidopsis* protein by sometimes up to 10‐fold (Figure 1B; Zand Karimi et al. 2025) and thus bias enrichments toward chloroplasts. In fact, for all three APF proteome datasets generated here, the Rubisco large subunit was the most abundant polypeptide detected in the MS1 scans with the small subunit often ranked close by (Datasets S2–S4), despite the samples containing few to no chloroplasts as judged by chlorophyll measurements (Figure 1A).

**FIGURE 2:**
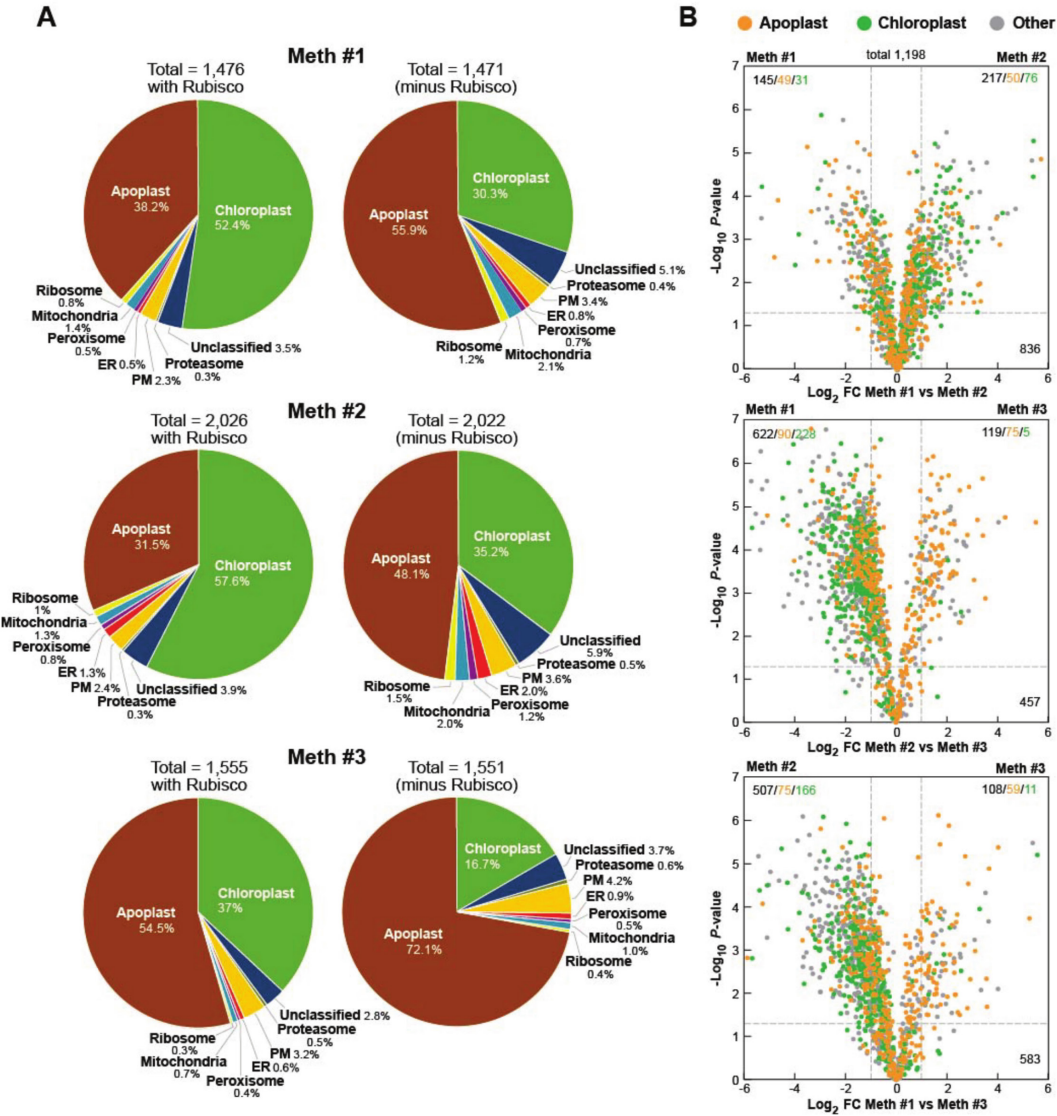
Proteomic comparisons of the *Arabidopsis* APF isolated by the three VIC methods. APF proteins, isolated as schematically illustrated in Figure S1, were identified by LC–MS/MS and normalized for abundance across datasets based on the combined MS1 precursor ion intensities of each sample. (A) Pie charts illustrating the proportional abundance of APF proteins assigned to specific subcellular compartments or protein complexes, as defined by GO terms and an apoplast protein catalog developed by Zand Karimi et al. (2025). The left pie charts were based on the combined MS1 intensities for all identified proteins, while the right pie charts were based on recalculated combined MS1 intensities after removing those derived from the Rubisco large and small subunits (5, 4, and 4 total polypeptides for Methods 1, 2, and 3, respectively). The percentages for each compartment/complex were based on the total precursor ion abundances calculated from the MS1 scans. (B) Volcano plots comparing the relative abundance of 1198 proteins in common obtained by the three isolation methods (minus Rubisco). The dashed lines indicate the significance boundaries based on both Log_2_ FC ≥ 1 or ≤ −1 and *p*‐value of significance < 0.05. Green and orange points identify chloroplast and apoplast proteins, respectively. All others are colored in gray. The numbers in the top left and right quadrants indicate the total number of proteins, and the apoplast, and chloroplast proteins with significant differences between samples. The bottom numbers reflect all those proteins with insignificant differences between samples.

Using this abundance measure based on MS1 scans, we reanalyzed the compartment/complex profiles generated by the three isolation methods either with the large and small subunits of Rubisco included or excluded. As seen from the pie charts in Figure 2A, the abundance of chloroplast proteins still dominated the three VIC samples with Rubisco included, with 52.4%, 57.6%, and 37% of the MS1 precursor ion intensities being assigned to peptides from this compartment in the respective Method 1, 2, and 3 samples. These chloroplast values then dropped substantially to 30.3%, 35.2%, and 16.7% simply by removing values for Rubisco (5, 4, and 4 Rubisco polypeptides among the 1471, 2022, and 1551 total polypeptides detected, respectively), demonstrating that this chloroplast dominance was primarily caused by Rubisco and not whole organelle contamination. By contrast, the prevalence of apoplast proteins based on MS1 scans rose dramatically without Rubisco from being the second most abundant compartment with Rubisco included to now dominating the respective pie charts with 55.9%, 46.1%, and 72.1% of combined protein abundances predicted to be apoplastic for the three isolation methods (Figure 2A).

This enrichment for apoplastic proteins as compared to those from other cellular compartments or complexes was similarly seen by bar charts analyzed by one‐way ANOVA with Tukey's post hoc statistical tests based on calculations of percent normalized abundance based on the MS1 scans with or without the inclusion of values from Rubisco (Figure S2). Catalogs generated by Method 3 had the highest percentage of apoplast proteins and the lowest percentages of proteins from chloroplasts, mitochondria, ribosomes, peroxisomes, and ER. Only proteins currently assigned to the plasma membrane were similarly enriched by Method 3, which could be explained by its proximity to the apoplastic space. Collectively, comparisons among the VIC methods further demonstrated that our improved Method 3 provided the best enrichment for apoplast constituents relative to other compartments.

To further emphasize the enrichment for apoplast proteins relative to chloroplast proteins using Method 3, we assessed the relative abundance of the 1198 proteins in common among the three VIC samples (with the exclusion of Rubisco polypeptides) by volcano plots comparing both Log_2_ FC ≥ 1 or ≤ −1 differences in abundance and *p* < 0.05. As illustrated in Figure 2B, Method 3 samples were best enriched for apoplast proteins versus those from chloroplasts, as shown by their distributions in the plots. In comparisons of samples derived from Method 3 versus Method 1 or 2, the catalog of 348 chloroplast proteins detected was significantly less abundant in Method 3 (5 versus 228 and 11 versus 166, respectively) while the 339 apoplast proteins detected were similarly abundant (75 versus 90 and 59 versus 75, respectively) (Figure 2B). By contrast, the distribution of chloroplast proteins in the volcano plots was nearly equal when comparing Methods 1 and 2, suggesting that both approaches were less efficient in eliminating chloroplast constituents.

Additional volcano plots separately analyzing proteins associated with other compartments/complexes supported this view (Figure S3). As compared to Methods 1 and 2, Method 3 samples were more depleted of proteins associated with chloroplasts, mitochondria, plasma membrane, peroxisomes, and ribosomes but were similarly distributed for apoplast‐designated proteins. Using the scatter of 49 ribosomal proteins detected as an example (Figure S3), the significantly distributed proteins from Method 1 versus Method 2 were minimal (7 and 9), while the distribution skews for Method 3 versus Method 1 or Method 2 were substantial (2 and 38 or 1 and 34, respectively). Taken together, Method 3 was even better at enriching for apoplast proteins with less contamination when assessed based on collective protein abundances and not numbers.

### 3.3 GO Analyses of the APF Enrich for Apoplast Terms

**FIGURE 3:**
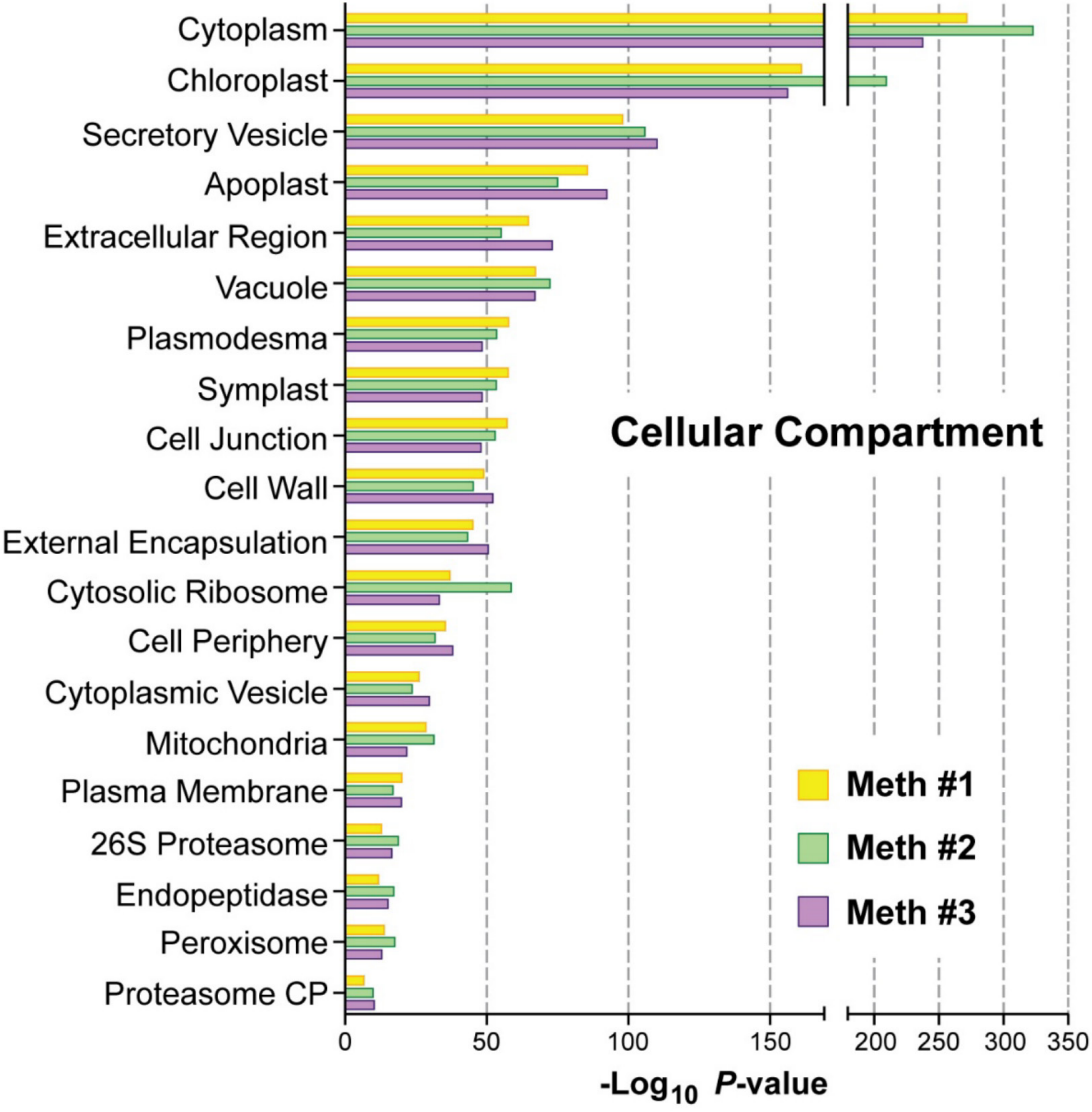
Gene Ontology (GO) enrichments based on the Cellular Compartment category for the collections of proteins identified by LC–MS/MS in samples derived from the three APF isolation methods. GO enrichment was performed with the g:Profiler platform using the 1471, 2022, and 1551 proteins from Methods 1, 2, and 3, respectively, after removing Rubisco polypeptides (Datasets S1–S3). The *p*‐values of enrichment were calculated using multiple testing corrections and application of the default g:GOSt algorithm to adjust for significance scores. Cellular Compartment terms were selected based on their uniqueness, statistical significance (*p*‐value), and overall completeness of annotation.

Gene Ontology (GO) analyses (https://biit.cs.ut.ee/gprofiler/gost) of the three VIC preparations further supported the dominance of apoplast functions in the APF preparations and a better enrichment for these functions by Method 3. As compared to those of Methods 1 and 2, GO Cellular Compartment terms commonly connected to the apoplast were more enriched by Method 3 with higher *p*‐values of significance for “secretory vesicle,” “apoplast,” “extracellular region,” and “cell wall” terms, but less enriched for terms related to intracellular compartments such as “cytoplasm,” “chloroplast,” “mitochondria,” and “peroxisome” (Figure 3). It is noteworthy that Method 2 samples were substantially more enriched for the “chloroplast” term (Figure 3), in agreement with its higher levels of chlorophyll and Rubisco as seen biochemically (Figure 1A–C).

Additional GO analyses using the Molecular Function and Biological Process categories further supported the superior concentration of apoplastic activities by Method 3. Included were better enrichments for “hydrolase,” “anti‐oxidant activity,” “exopeptidase,” “peptidase,” and “glucosidase” activities, “carbohydrate metabolism,” “cell wall,” “macromolecular catabolic,” “response to biotic stimulus,” and “defense responses” terms consistent with the strong hydrolytic activity, cell wall dynamic, and pathogen defense processes associated with the apoplast (Figure S4). Again, Method 2 samples were better enriched for terms associated with contaminating intracellular activities, including “ribosome,” “mRNA binding,” “nucleotide metabolism,” and several intracellular metabolic pathways and processes (e.g., “glycolysis,” “photosynthesis,” and “translation”) (Figure S4).

### 3.4 Subunits of the 26S Proteasome Can Be Found in the APF

**FIGURE 4:**
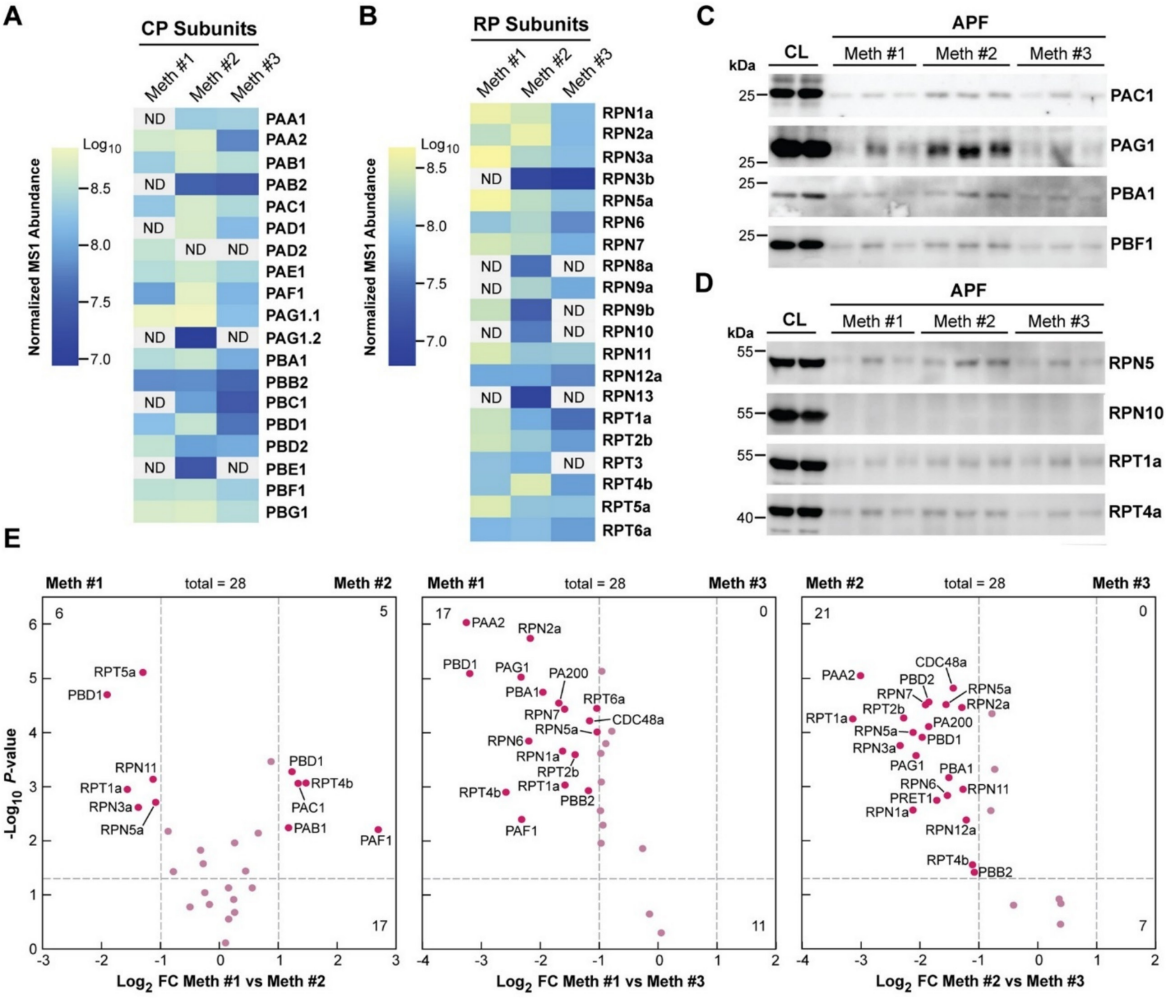
APF isolation methods recover subunits of the 26S proteasome. Comparison of 26S proteasome subunit levels found enriched by the three APF isolation methods. APF isolates were analyzed by LC–MS/MS and immunoblotting for subunits of the CP and RP subcomplexes. (A and B) Quantitative MS/MS detection of the 14 CP subunits (A) and 12 of the 18 RP subunits (B) by heat maps normalized by precursor ion abundances in the MS1 scans. MS/MS values reflect the average of the three biological replicates, each with two technical replicates. The two splice isoforms of PAG1 were detected only in Method 2 samples while the shorter isoform (PAG1.1) was detected by all three methods. (C and D) Immunoblot analysis for specific CP (C) and RP subunits (D) in the APF samples following SDS‐PAGE. TL samples and the APF extracted by all three methods (10‐ and 15‐μg total protein, respectively) were added to the lanes. Two and three independent batches were assayed for total leaf lysate (TL) and APF, respectively. (E) Volcano plots showing the relative distribution of proteasome subunits found in common among samples isolated by the three methods (total = 28). Individual subunits (colored in red) showing differences in abundance are labeled. The numbers in the top left and right quadrants indicate the total number of proteasome subunits with significant differences between the APF samples based on both Log_2_ FC ≥ 1 or ≤ −1 and *p*‐values of significance < 0.05. The bottom numbers reflect all those proteins with insignificant differences between samples.

One impetus for developing an improved VIC strategy for APF isolation was to support our recent discovery that proteasomes central to ubiquitin‐mediated proteolysis accumulate in the extracellular space (Zand Karimi et al. 2025). In fact, 50‐μM ATP was specifically included in the APF infiltration solutions to protect this particle from dissociation (Book et al. 2010). As shown by the LC–MS/MS analyses in Figure 4A,B, all three VIC strategies enriched for proteasomes with most, but not all, 26S particle subunits detected. These included all 14 polypeptides needed to assemble the 20S core particle (CP) that compartmentalizes the protease activity, and many of the 19 polypeptides needed to assemble the 19S regulatory particle (RP) that helps with substrate selection/unfolding and ubiquitin recycling (Book et al. 2010; Russell et al. 2013). The overall MS/MS coverage and detection of the subunits were roughly equivalent among the isolation strategies. For the PAG1 subunit, both splice variants were detected by Method 2 while only the shorter variant (PAG1.1) was found by the other two methods.

We also easily detected various RP and CP subunits by immunoblot analyses of the APFs using antibodies against the CP subunits PAC1, PAG1, PBA1, and PBF1 or against the RP subunits RPN5, RPT1a, and RPT4a (Figure 4C). Consistent with most 26S proteasomes accumulating in the nucleus and cytoplasm (Book et al. 2010), the immunoblot signals were substantially stronger in the total lysate versus the APFs. Volcano plot comparisons of the proteasome subunits in common among the three VIC datasets also found stronger enrichment for these subunits in Methods 1 and 2 consistent with their higher levels of intracellular contamination. As seen previously (Zand Karimi et al. 2025), we typically failed to detect RPN10 in all three APF samples by immunoblotting and poorly detected it by LC–MS/MS even though this RP subunit was easily detected in the total lysates (Figure 4). Its absence suggests that a unique proteasome type accumulates in the apoplast.

### 3.5 Aggregation of the APF Datasets Reveals a Core Apoplast Proteome

**FIGURE 5:**
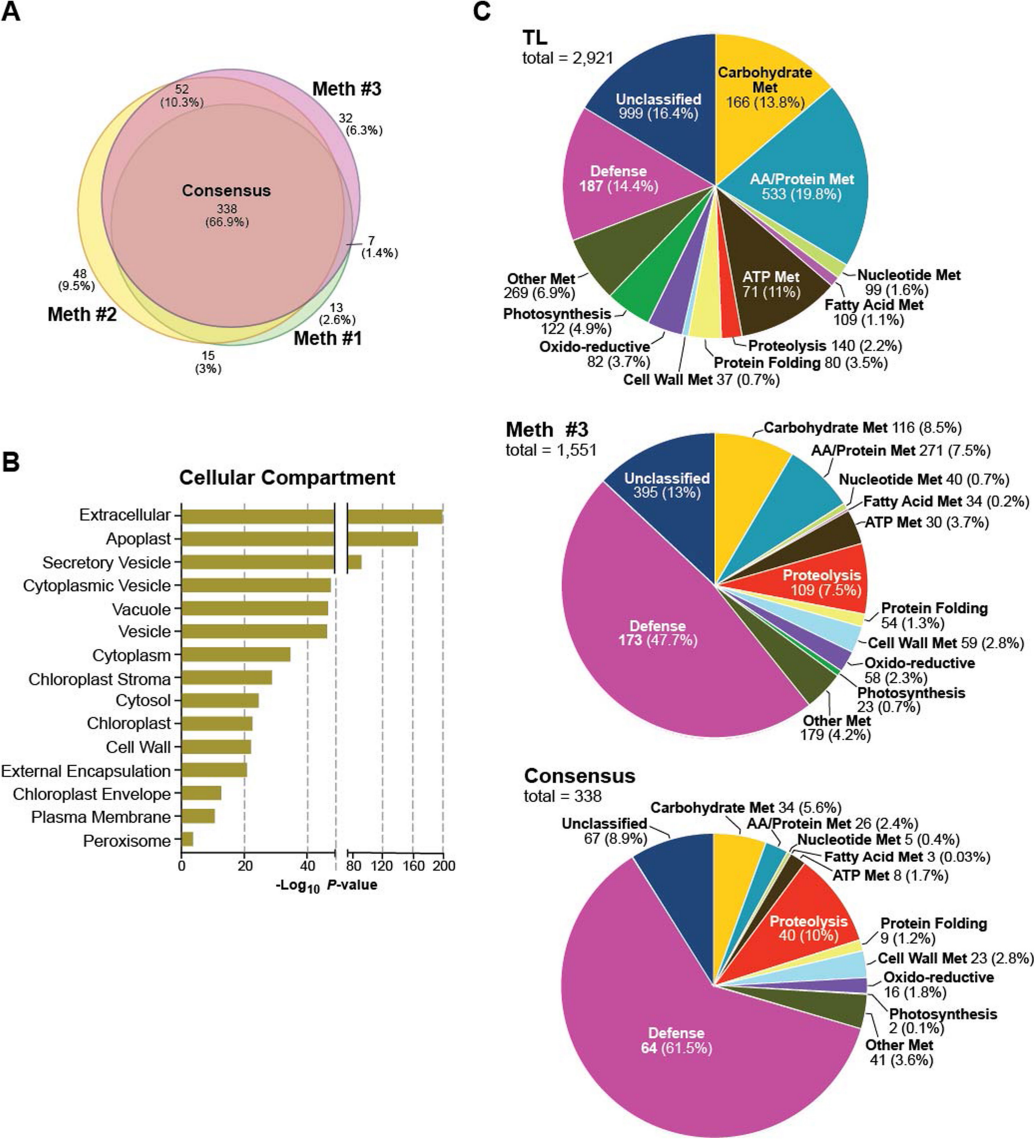
Consensus core proteome of the Arabidopsis APF reveals a strong enrichment for apoplast functions. (A) Venn diagram revealing the 338 consensus apoplast/extracellular proteins, as defined by Gene Ontology (GO), which were identified by LC–MS/MS1 as in common among the three methods (373, 453, and 429 for Methods 1, 2, and 3, respectively; Figure 1G). (B) Gene Ontology (GO) enrichments for terms within the Cellular Compartment category for the consensus collection of APF proteins. The *p*‐values of enrichment were calculated using multiple testing corrections and the application of the default g:GOSt algorithm to adjust for significance scores. (C) Pie chart comparisons based on the Biological Function category in GO for the total number of proteins (minus Rubisco polypeptides) identified by LC–MS/MS in the total leaf lysate (TL; 2921 total), the APF isolated by Method 3 (1551 total), and the consensus APF proteome generated with apoplast/extracellular proteins found in common for all three isolation methods (338 total). The numbers represent the total number of proteins for each term, and the percentage was calculated using the combined MS1 intensities after removing the MS1 intensities derived from Rubisco.

In an attempt to identify core components of the apoplast, we generated a consensus proteome using the protein collections (minus Rubisco) from the three VIC preparations that were assigned by GO to the “APF/extracellular” term (Figure 1G). By comparison of the 373, 453, and 429 proteins with this designation, we identified 338 proteins in common whose GO classifications based on the Cellular Compartment category strongly fit an apoplastic location (Figure 5A,B). The top GO terms for this collection based on *p*‐values for enrichment were for “extracellular,” “apoplast,” and “secretory vesicle,” as expected for proteins found within the apoplast with substantially higher *p*‐value for various intracellular compartments such as “cytoplasm,” “chloroplast,” and “peroxisome” thought to be contaminants (Figure 5B).

We then compared the consensus apoplast catalog against those proteins detected by LC–MS/MS in the total lysate and in the total APF catalog isolated by Method 3 (minus Rubisco). As seen by the pie charts based on GO in Figure 5C, we saw a robust concentration of activities associated with various apoplast function terms when comparing the total lysate, APF, and consensus APF by aggregated MS1 precursor ion abundances. For example, proteins assigned by GO to “defense” rose from 14.4% of the cumulative MS1 ion intensities in the total lysate, to 47.7% of the APF, and finally to 61.5% of the consensus APF. By comparison, proteins assigned to photosynthesis declined from 4.9% of the total lysate, to 0.75% of the APF, and finally to 0.1% of the consensus APF as an indicator of diminishing chloroplast contamination. Other Biological Function terms that significantly changed in abundance as apoplastic proteins presumably became more enriched, included “proteolysis,” which rose from 2.2% to eventually 10%, and “cell wall metabolism,” which rose from 0.7% to eventually 2.8%, whereas proteins associated with “amino acid/protein metabolism” commonly associated with intracellular activities conversely fell from 19.8% eventually to 2.4% (Figure 5C). Several other GO terms failed to change substantially upon APF enrichment (e.g., “carbohydrate metabolism,” nucleotide metabolism, and “oxido‐reductive”), which likely reflected apoplastic activities also found in intracellular compartments.

Altogether, we concluded in agreement with prior studies (see above), that the core, likely abundant proteins in the APF drive activities related to plant defense, proteolysis, and cell wall synthesis and dynamics. This enrichment was especially obvious within the top 50 proteins in the consensus catalog, which included numerous PR proteins (e.g., PR1, PR2, and PR5), a collection of α‐ and *β*‐glycosidases and glucosyl hydrolases, chitinases, pectin esterases, xylosidases, lipases, and aspartyl and subtilisin‐type serine proteases (Table 1). These top 50 apoplast candidates were within the top 77 proteins identified in the Method 3 dataset as well as also being abundant in the Method 1 and 2 datasets (Datasets S2–S5). Interestingly, many APF proteins have direct connections to biotic defense and/or were found to be upregulated by hydrogen peroxide or upon pathogen attack (58%; Table 1), consistent with roles in ROS production and microbial defense (Bindschedler et al. 2008; Jiang et al. 2023).

**TABLE 1:** Top consensus apoplastic proteins in the arabidopsis leaf APF.^a^

Chromosome location	Protein name	Function/activity	SP/TP^b^	Rank^c^	Def.^d^
At1g75040.1	PR5	Pathogenesis related‐5/thaumatin‐like	SP	1	+
At3g57260.1	BGL2/PR2	Pathogenesis related‐2/β‐1‐3 glucanase‐2	SP	2	+
At3g57240.1	BG3	β‐1‐3 Glucanase‐2	SP	3	+
At2g43570.1	CHI	Chitinase	SP	5	+
At1g09750.1	AED3	EDS1‐dependent aspartyl protease‐3	SP	6	+
At5g10760.1	AED1	EDS1‐dependent aspartyl protease‐1	SP	7	+
At2g14610.1	PR1	Pathogenesis related‐1, SA‐responsive	SP	8	+
At1g76160.1	SKS5	Cu‐oxidoreductase	SP	10	+
At5g67360.1	SBT1.7	Subtilisin‐like Ser protease	SP	13	+
At5g13980.2		α‐Mannosidase (family 38)	SP	15	
At1g21670.1		DPP6 domain containing	SP	16	
At5g64570.1	XYL4	β‐D‐Xylosidase‐4 (family 3 glycoside hydrolase)	PS	17	+
At5g08380.1	AGAL1	α‐Galactosidase‐1	SP	22	
At3g18490.1	ASPG1	Aspartyl protease in guard cells‐1	SP	24	
At3g52840.2	βGAL2	β‐Galactosidase‐2	None	25	
At4g20840.2	BBE21	Oligogalacturonide oxidase‐2 (berberine family)	SP	27	+
At2g28470.1	βGAL8	β‐Galactosidase‐8	SP	28	
At5g55450.1	LTP4	Bifunctional inhibitor/lipid transfer‐4	SP	30	+
At5g04140.1	GLU1	Glutamine synthase‐1	cTP	31	
At1g29660.1	GGL5	GDSL‐motif esterase/acyl transferase/lipase	SP	32	+
At3g14210.1	ESM 1	Glucosinolate hydrolase	SP	33	+
At3g62030.2	ROC4	Rotamerase CYP‐4/cyclophilin CYP‐20.3	mTP	35	+
At2g10940.1		Bifunctional inhibitor/lipid transfer	SP	38	
At1g79720.1		Aspartyl protease	SP	41	
At4g27520.1	ENODL2	Early nodulin‐like‐2	SP	42	
At1g23310.1	GGT1	Glutamate:glyoxylate aminotransferase‐1	None	45	
At3g14415.3	GOX2	Glycolate oxidase‐2	None	46	+
At2g21330.1	FBA1	Fructose bisphosphate aldolase	cTP	47	
At4g33010.1	GLDP1	Glycine decarboxylase P‐protein‐1	cTP	50	
At3g54050.1	CFBP1	Fructose 1,6‐bisphosphate phosphatase	cTP	51	
At5g02500.1	HSC70‐1	Heat shock protein 70–1	None	52	+
At3g11630.1	2CPA	2‐Cys peroxiredoxin	cTP	54	+
At1g04410.1	NDH1	NAD‐dependent malate dehydrogenase	None	55	+
At3g07390.1	AIR12	Auxin‐induced in Root Culture‐12	SP	56	
At1g78830.1	CCLP	Cucurlin‐like lectin family	SP	57	
At2g46930.1	PAE3	Pectin acetyltransferase‐3	SP	58	
At3g55440.1	TPI	Triosephosphate isomerase	None	59	+
At1g68010.2	HPR	Hydroxypyruvate reductase	None	60	
At2g13360.1	AGT1	Alanine/serine:glycolate aminotransferase	None	63	
At1g09340.2	CRB	Chloroplasts RNA‐binding protein	None	64	+
At2g45470.1	FLA8	Fasciclin‐like arabinogalactan protein‐8	SP	65	
At2g36530.1	ENO3	Enolase‐2	None	67	+
At5g06860.1	AGIP1	Polygalacturonase‐inhibiting protein	SP	69	+
At1g65930.1	cICDH	NADP^+^ dependent isocitrate dehydrogenase	None	71	+
At3g14420.2	GOX1	Glycolate oxidase‐1	None	72	+
At2g38540.1	LTP1	Lipid transfer protein‐1/PR14‐like	SP	73	
At5g47550.1	CYS5	Cystatin proteinase inhibitor‐5	SP	74	
At1g19570.1	DHAR1	Dehydroascorbate reductase‐1	None	75	+
At1g66970.1	GDPGL1	Glycerolphosphodiester phosphodiesterase‐like1	SP	76	
At5g11720.1	AGLU1	α‐Glucosidase‐1 (family 31)	SP	77	

^a^ Top 50 ranked consensus apoplast proteins found in common among the three APF datasets ranked in abundance as determined using MS1 precursor ion intensities derived from LC–MS/MS.

^b^ Predicted presence of N‐terminal sequences for either signal peptides (SP) or mitochondrial (mTP) or chloroplastic transit peptides (cTP). None, none predicted.

^c^ Abundance rank in the Method 3 APF dataset based on MS1 precursor ion counts.

^d^ +, linked previously to biotic defense.

### 3.6 Protein Sequence Analyses of APF Proteins Suggest Multiple Routes for Secretion

**FIGURE 6:**
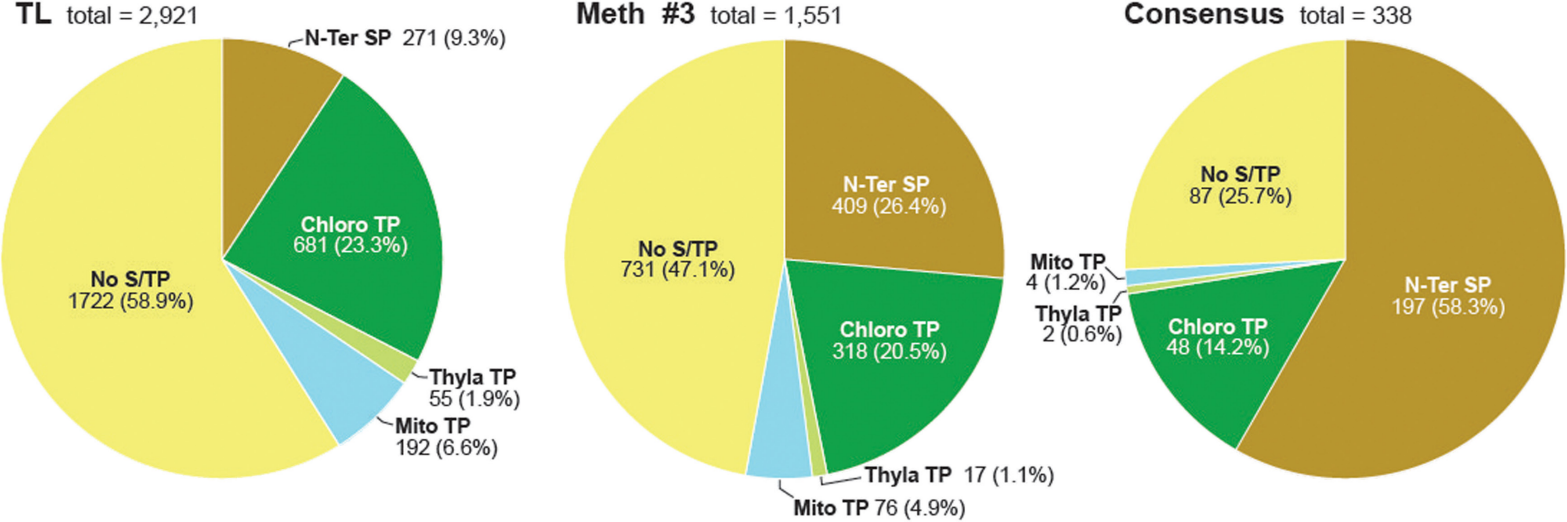
Distribution of proteins in the APF catalogs predicted to harbor N‐terminal cellular transport sequences. Amino acid sequences from the protein catalogs identified here by LC–MS/MS from total leaf lysates, the APF isolated using Method 3, and the consensus APF catalog generated by the overlapping protein profiles obtained by the three APF isolation methods were analyzed for possible signal (SP) or transit peptide sequences (TP) using TargetP2.0 (Almagro Armenteros et al. 2019). Shown are pie charts illustrating the numbers and percentages of proteins in each collection that were predicted to have no obvious signal/transit peptide sequence (No‐S/TP), those with predicted N‐terminal SPs for the ER (N‐Ter SP), and those with N‐terminal transit peptides for the chloroplast (Chloro‐TP), chloroplast thylakoid lumen (Thyla‐TP), and mitochondria (Mito‐TP).

Secretion of plant apoplastic proteins is likely a complex process involving multiple routes (Delaunois et al. 2013; Rodriguez‐Celma et al. 2016), including the classical ER to Golgi to apoplast export pathways that use N‐terminal signal peptides for entry, and poorly understood, non‐classical routes possibly without signal peptides, some of which involve multi‐vesicular bodies, secretion through vacuole/plasma membrane fusion, and exocyst‐positive organelle(s) (EXPO) (Tang et al. 2024). To help discern how apoplastic proteins might be secreted, we scanned the consensus APF and Method 3 APF catalogs for possible signal/transit sequences using TargetP2.0 (https://services.healthtech.dtu.dk/services/TargetP‐2.0/), a machine‐learning algorithm that searches for possible N‐terminal motifs directing proteins into the ER, mitochondria, chloroplasts, and the thylakoid lumen (Almagro Armenteros et al. 2019; Emanuelsson et al. 2007). In agreement with the protein distribution of the entire *Arabidopsis* proteome, most proteins detected in the total leaf lysates were devoid of such signal/transit peptides (58.9%), with the next most abundant collection predicted to harbor chloroplast transit peptides (23.3%) (Figure 6). Only a few had a possible signal peptide sequence (9.3%). By contrast, the percentages of apoplast proteins identified by Method 3 with no obvious ER signal sequence or containing a chloroplast transit peptide sequences dropped substantially as compared to the total lysate (47.1% and 20.5% respectively), while those with an N‐terminal ER signal sequence rose to 26.4%, implying that APF enrichment also enriched for proteins subject to classical ER export mechanisms while removing chloroplast‐ and mitochondria‐targeted proteins (Figure 6).

Strikingly, when we analyzed the consensus APF catalog, those with a predicted N‐terminal signal peptide sequence rose again to 58.3%, with the top 14 proteins all forecast to have this motif (Figure 6 and Table 1). By contrast, those predicted to harbor transit sequences for other compartments dropped to less than 16% of the total (Figure 6). This drop was especially strong for polypeptides with predicted mitochondria or thylakoid lumen transit peptides (only 1.2% and 0.6%, respectively), further underscoring the strong enrichment for apoplastic proteins and loss of proteins destined for other compartments. Conversely, proteins without discernible transit sequences (also called leaderless secreted proteins; Delaunois et al. 2013) remained high (25.7%), suggesting that many apoplast proteins use non‐classical routes for extracellular transport (Figure 6).

We also analyzed the APF datasets using ApoplastP (Sperschneider et al. 2018), a tool that specifically predicts proteins destined for the apoplast. While the results were generally consistent with those generated by TargetP 2.0, far fewer proteins were predicted to be apoplastic, with only 4.8%, 11.7%, and 28.2% predicted to be secreted in the total leaf lysate, the Method 3 APF, and the consensus APF, respectively.

## 4 Discussion

Despite its importance to numerous physiological and developmental processes in plants and its crucial roles in defense against a range of environmental insults (Martinez‐Gonzalez et al. 2018; Farvardin et al. 2020), the apoplast remains poorly defined mainly due to challenges to its extraction free of intracellular contaminants. Here, we attempted to enhance understanding through a streamlined VIC isolation protocol for *Arabidopsis* leaves coupled with improved proteomic analysis by LC–MS/MS (Zand Karimi et al. 2025). By simultaneous comparison with two current VIC approaches using identical tissue, biochemical assays, and LC–MS/MS analyses (Huang et al. 2021; Rutter et al. 2017), we found that our method (Method 3) best minimized cytoplastic contamination, while still generating deep APF catalogs. The likely reasons for the improvement as compared to Method 2 were the use of leaves alone that were infiltrated under low pressure and centrifugally extracted under lower *g*‐forces (Lohaus et al. 2001) without bundling and with the cut petiole ends pointing up (Figure S1B; Huang et al. 2021). Presumably, the leaf orientation minimized tissue damage and forced infiltrate egress through stomata and hydathodes and not the cut site and phloem, with the added advantage of providing extra filtration before exit. By contrast, Method 1 might be compromised by packing 4–6 whole rosettes into the collection tubes which could increase tissue damage during centrifugation. We also note the addition of 50 μM ATP to the common infiltrate solution given that this nucleotide is found at reasonably high concentrations within this space (Song et al. 2006).

The resulting APF catalog of ~1500 possible members identified by Method 3 now provides one of the most extensive views of the *Arabidopsis* APF proteome. As in previous, more limited studies (Huang et al. 2021; Boudart et al. 2005; Jiang et al. 2023; Borniego et al. 2019; Rutter and Innes 2017), we found a varied mix of hydrolytic activities against proteins, carbohydrates, lipids, and primary metabolites, making it a particularly hostile environment that likely compromises many intracellular processes while also antagonizing invasion by pathogens. In fact, many proteins related to defense (61.5%) and a number of pathogen‐related (PR) proteins upregulated during PTI defense (Bindschedler et al. 2008) were abundant in the APF datasets (Figure 5C and Table 1). This abundance was observed despite the use of healthy, non‐infected leaves, suggesting that much of the PTI‐associated proteome is constitutively present. We also found proteinaceous inhibitors (e.g., cystatin (At5g47550.1), the polygalacturonase inhibitor AGIP1 (At5g06860.1), and the lipid transfer protein‐4 (LTP4; At5g55450.1) classified as a bifunctional inhibitor (Table 1) in the APF, which could mute the activit(ies) of extracellular host hydrolases as well as those from pathogens.

Furthermore, when all the *Arabidopsis* LC–MS/MS datasets generated here, involving nine biological replicates each analyzed twice (18 total), were combined, 1198 common constituents were identified (minus Rubisco), which represent a two or more fold deeper APF catalog as compared to prior MS analyses (Jiang et al. 2023; Bindschedler et al. 2008; Borniego et al. 2019; Boudart et al. 2005; Chaya et al. 2024), thus expanding the possible functions of this extracellular space. In a search for the core, abundant APF components, we also generated a consensus catalog of 338 likely abundant proteins compiled from the three methods based on apoplast assignments in TAIR. Consequently, we propose that the new APF protein lists generated here supplant prior assignments in TAIR for a more accurate view of the *Arabidopsis* apoplast proteome.

One of the main historical challenges to defining the apoplast proteome has been dealing with contamination from the cytoplasm. While our new VIC method does help, we acknowledge that it remains unclear which proteins in the expanding catalog are true intracellular contaminants as opposed to proteins that were misassigned to cytoplasmic compartments or those that reside both internally and externally. It should also be appreciated that some of the “contaminants” detected here in the APF samples might represent cytoplasmic constituents deliberately exported to the apoplast for extracellular turnover given the plethora of proteases in the APF, and/or their release into this space during unavoidable cell leakage or following programmed cell death of specific cell types as plant tissues mature (e.g., xylem and sclerenchyma). Clearly, localization studies through cell biological approaches will be needed to confirm the actual location of these currently proposed “cytoplasmic” polypeptides also found in the APF.

Surveys of the APF protein catalogs revealed a plethora of activities presumably important to apoplast functions. Included are glycosidases, chitinases, and various proteases that serve not only to attack pathogens directly but also to aid the release cell wall fragments and peptides from the pathogens as part the host PTI surveillance systems that then recognize these fragments as microbe‐associated molecular patterns (MAMPs) (Martinez‐Gonzalez et al. 2018). One example involves extracellular proteasomes (Figure 5) along with the subtilases SBT1.7 (At5g73360) and SBT5.2 (At1g20160), and likely a collection of endo‐1‐3‐β‐glycosidases (PR‐2/*β*‐1,3‐glucanse‐2 [At3g57260], *β*‐GAL2 [At3g52840], and *β*‐GAL‐8 [At2g28470]) that work in concert to release the naked 22‐amino‐acid flg22 MAMP from the bacterial flagellin glycoprotein (Zand Karimi et al. 2025; Buscaill et al. 2019; Matsui et al. 2024). flg22 then triggers a PTI response involving ROS after recognition by the plasma membrane‐bound flg22 receptor FLS2. It is noteworthy that the proteins encoding these three activities are commonly found in the APF (Dataset S4), with several estimated to be highly abundant based on MS1 precursor ion intensities (Table 1).

The APF also contains a number of enzymes related to ROS, including peroxidases, thioredoxins and 2‐Cys periredoxins, catalase‐1, monodehydroascorbate reductase‐1, dehydroascorbate reductase, and glycolate oxidase‐2 that regulate ROS levels involved in pathogen defense and cell wall strengthening (Table 1 and Dataset S5) (Mata‐Perez and Spoel 2019; Pelaez‐Vico et al. 2024). Also abundant were the thaumatin‐like protein PR‐5 (At1g75040.1), whose levels rise upon salicylic acid and pathogen exposure and during senescence, and appears to provide anti‐fungal and anti‐freeze activities (Feng et al. 2024; Borniego et al. 2019; Jiang et al. 2023), and the PR‐2 (At3g57260) and BGL2 (At2g43570) β1–3 glucanases, and the CHI chitinase (At3g57240), which could help release oligosaccharine fragments as MAMPs (Table 1), as well as deter infection by attacking the pathogen's exterior. Notably, these above‐mentioned proteins were typically the most abundant APF constituents outside of Rubisco in our three APF datasets (Table 1 and Datasets S2–S5). Similarly, the ZYL4 xyloglucanase within the endo/transglycosidase/hydrolase (XTH) superfamily (At5g64570), the pectin acetylesterase PAE3 (At2g46930), and the GGL5 GDSL lipase (At1g29660) are abundant in the consensus APF (Table 1). They presumably enhance disease resistance by degrading pathogen components, while another abundant APF protein, FAD‐binding Berberine BBE21 (At4g20840), is predicted to oxidize oligogalacturonides, thus helping disassemble fungal cell walls. It should be emphasized that the apoplastic destination for many of these PTI‐related defense proteins was expected given that pathogens initially contact their plant hosts through this extracellular space (Darino et al. 2022; Ngou et al. 2022).

Our APF catalogs also included most, if not all, CP and RP subunits that comprise the 26S proteasome (Figure 4), further supporting the export of this proteolytic complex into the apoplast (Zand Karimi et al. 2025). We presume that the detection of both subcomplexes, especially the RP, was aided by adding ATP to the infiltration fluid to maintain 26S particle stability. Our subsequent searches of other publicly available APF databases derived from several plant species but without using ATP during extraction also identified a similar but typically more limited collection of proteasome polypeptides (Ceballos‐Laita et al. 2015; Grosse‐Holz et al. 2018; Jiang et al. 2023; Carella et al. 2016; Abdul Haseeb et al. 2022). As seen with our MS/MS analyses (this report; Zand Karimi et al. 2025), coverage of CP subunits in these prior reports often exceeded those of the RP (Table S1), supporting our expectation that the CP subcomplex harboring the proteasome proteolytic sites is more prevalent. The absence of the Rpt1–6 subunits needed for substrate unfolding by the RP was particularly notable.

As seen previously in various APF proteomic studies (e.g., Zand Karimi et al. 2025; Bindschedler et al. 2008; Jiang et al. 2023; Rodriguez‐Celma et al. 2016), the Rubisco large and small subunits were universal, highly abundant members of our APF datasets. In fact, their levels were sufficiently high to skew proteome compartment profiles based on MS1 precursor ion intensities toward chloroplasts despite having minimal impact on protein numbers (see Figures 1G and 2A). While their prevalence was consistent with contamination based on correlation and *R* ^2^ values comparing the three methods, alternative explanations include their deliberate secretion as part of an autophagic process for Rubisco recycling (Zhuang and Jiang 2019) or their unavoidable release into the apoplast during programmed cell death as the leaf matures. It is noteworthy that other “chloroplast” proteins were also present to a lesser extent in our APF catalogs (Table 1 and Datasets S2–S4). While their inclusion could also reflect contamination, another possible interpretation is that at least some of these proteins actually reside in multiple compartments including the apoplast. Glutamate synthase‐1 (At5g04140.3), alanine/serine glyoxylate aminotransferase (At1g23310.1), and hydroxypyruvate reductase (At1g68010.2) are three such examples, which has been found in several intracellular compartments besides the apoplast (TAIR: https://www.arabidopsis.org) (Bindschedler et al. 2008; Taira et al. 2004). An intriguing hypothesis is that these extracellular versions aid primary metabolism outside the cell (e.g., external ammonia assimilation), metabolite recycling after release by external catabolic reactions, and/or niche management for beneficial microorganisms. Extracellular ATP might also help in these reactions.

An unanswered question is how apoplastic proteins are directed outside the cell after translation inside. Searches for secretion signals based on polypeptide sequence via TargetP2.0 (Almagro Armenteros et al. 2019) and ApoplastP (Sperschneider et al. 2018) suggested that most APF proteins use classical signal peptide sequences at their N‐terminal to direct ER to Golgi to apoplast transport. However, a substantial percentage failed these predictions (25.7% for TargetP2.0) such as proteasome subunits, suggesting that non‐classical “leaderless” routes are also common (Tang et al. 2024). At present, the mechanism(s) behind this leaderless secretion are not well known. At least for proteasomes, it does not appear to involve the canonical, ATG8‐dependent autophagic machinery nor an amphisome intermediate vesicle based on the proteomic analysis of APF catalogs derived from related mutants (Zand Karimi et al. 2025). Clearly, it is now of great interest to define not only these non‐classical routes but also how the classical route directs the ER‐localized polypeptides to the apoplast specifically and not to other internal compartments. It is also unclear how multi‐subunit proteasomes, likely fully assembled in the cytoplasm (Gemperline et al. 2019), end up in the apoplast. Not surprisingly, the probability of apoplast secretion of individual proteasome subunits by Neural Network (NN) scores through SecretomeP 2‐0 (Bendtsen et al. 2004) also did not predict a classical secretion route (Table S1).

Despite our and others progress in defining the *Arabidopsis* APF proteome (e.g., this report; Zand Karimi et al. 2025; Delaunois et al. 2013; Song et al. 2011; Grosse‐Holz et al. 2018; Jiang et al. 2023), we acknowledge that full understandings of the plant extracellular space remain challenged, mostly by difficulties in extracting large volumes of this fluid from intact tissues without tissue damage or cytoplasmic contamination. It is also likely that we missed anchored proteins or those too large to escape the tangled cell wall; adding robust proteases (e.g., trypsin) to the infiltrate solution might help release MS‐detectable peptides from these recalcitrant polypeptides. Possible improvements outside of VIC could involve laser‐capture microdissection in planta followed by LC–MS/MS (Zhu et al. 2016; Ezzoukhry et al. 2018), but additional progress is needed for subcellular descriptions. Alternatives might include exploiting mutants to genetically interrogate apoplastic secretion via the classical and non‐classical export routes (Tang et al. 2024) and/or developing improved computational algorithms beyond TargetP2.0 (Almagro Armenteros et al. 2019) and ApoplastP (Sperschneider et al. 2018) that can better predict apoplast‐specific deposition by sequence. For the latter strategy, the deeper APF lists presented here might help in designing more accurate apoplastic transport predictors via machine learning.

## Author Contributions

Kuo‐En Chen and Richard D. Vierstra designed the research. Kuo‐En Chen and Marilee Karinshak isolated the APF and total lysate samples and performed the biochemical assays, immunoblots, and chlorophyll measurements. Kuo‐En Chen performed the LC–MS/MS analysis, subsequent proteome analyses, and the bioinformatic search for signal/transit peptide sequences. Kuo‐En Chen and Richard D. Vierstra wrote the paper. All authors edited and approved the manuscript before submission.

## Conflicts of Interest

The authors declare no conflicts of interest.

## Peer Review

The peer review history for this article is available in the Supporting Information for this article.

## Supporting information


**Data S1.** Peer review.

**Dataset S1.** List of total proteins detected in apoplast fluid (APF) from all three VIC methods.
**Dataset S2.** List of proteins identified using Method 1 and their normalized abundances.
**Dataset S3.** List of proteins identified using Method 2 and their normalized abundances.
**Dataset S4.** List of proteins identified using Method 3 and their normalized abundances.
**Dataset S5.** List of consensus proteins in apoplast fluid (APF) common to all three VIC methods.

**Figure S1.** Comparisons of the current VIC protocols for APF isolation from *Arabidopsis* leaves.
**Figure S2.** Comparisons of enrichment for *Arabidopsis* leaf proteins assigned to various cellular compartments/complexes following isolated by the three APF enrichment methods.
**Figure S3.** Volcano plots showing the enrichment for various cellular compartments/complexes by the three APF isolation methods.
**Figure S4.** Gene Ontology (GO) enrichments based on the Molecular Function and Biological Process categories for the collection of proteins identified by LC–MS/MS in samples derived from the three APF isolation methods.
**Table S1.** Identification of proteasome subunits in previously published MS studies on the plant APF.

## Data Availability

The proteome datasets generated by this study have been deposited to the ProteomeXchange Consortium via the PRIDE partner repository and are available under the accession code PXD060844. Processed proteomic datasets are available in Datasets S1–S5.
