# Supplementary material for: Streamline Protocol for Arabidopsis Apoplastic Fluid Isolation Enables a Detailed Proteomic View of the Plant Extracellular Space
Source: Plant Direct. 2025 Jul 2;9(7):e70087. doi: 10.1002/pld3.70087 (PMC12222183; doi:10.1002/pld3.70087)

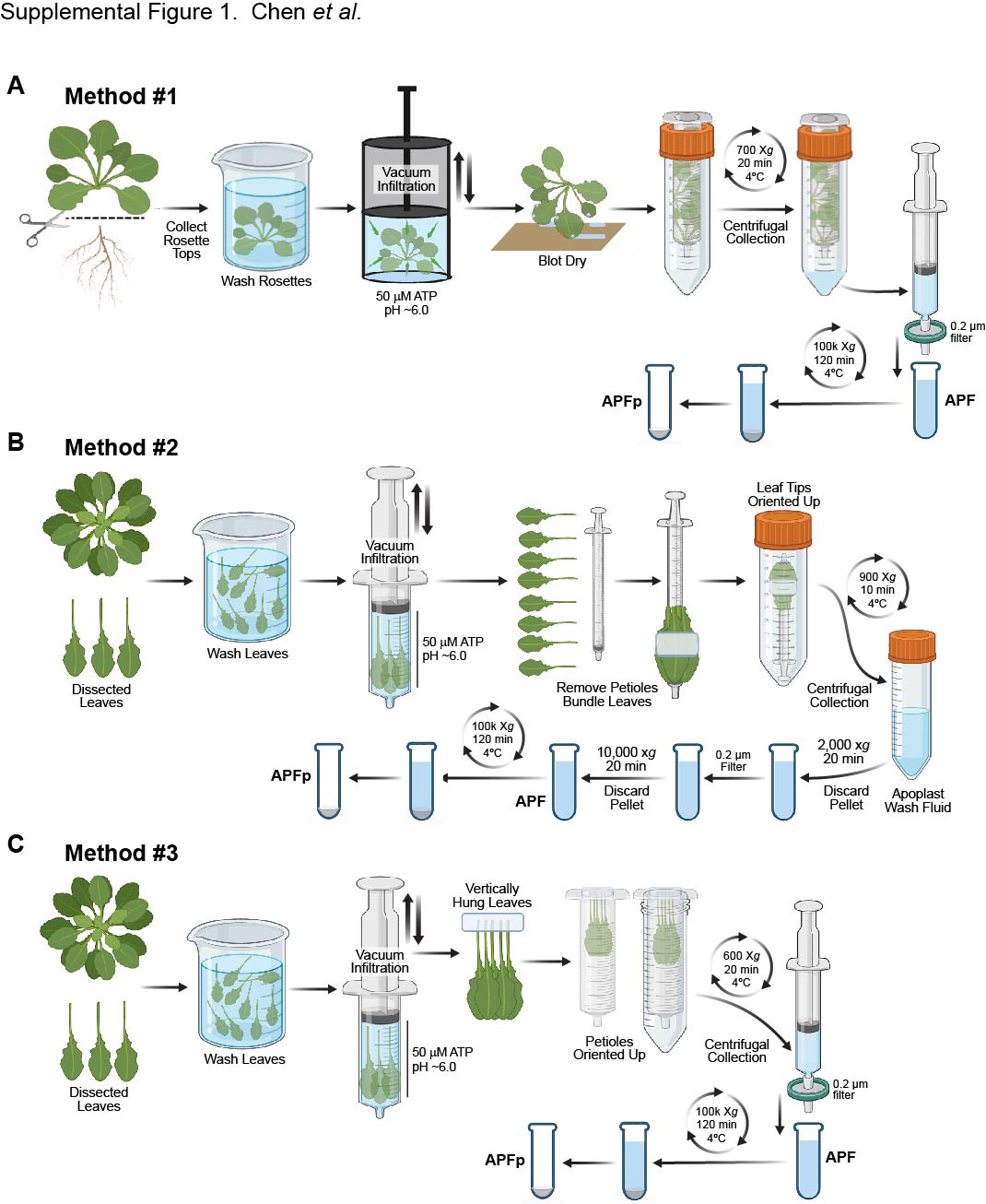


**Supplementary Figure S1│**Comparisons of current VIC protocols for APF isolation from Arabidopsis leaves. Shown are methods previously described by Rutter et al. (2017a) (Meth #1) and Huang et al. (2021) (Meth #2) along with the improved method described in detail here (Meth #3) (Zand-Karimi et al. 2025). To help stabilize proteasomes, 50 μM ATP was included in all extraction buffers. (A) Method #1 (Rutter et al., 2017a) vacuum infiltrates intact rosettes (minus roots) with APF extraction buffer, followed by blotting to remove excess fluid and collection of the infiltrated fluid by low-speed centrifugation (700 Xg) after packing into the collection tube. The fluid was clarified with a 20 μm cutoff filter to generate the APF. (B) Method #2 from Huang et al., (2021) dissects leaves from the rosettes by cutting at the stem/petiole junction, vacuum infiltrating the leaves with APF extraction buffer, blotting dry the leaves, removing the petioles, and bundling the leaves around a stick. The infiltrate was collected by low-speed (900 Xg) centrifugation of the bundle with the tips of the leaves oriented up. The fluid was first clarified by low-speed centrifugation (2,000 Xg), filtered through a 20-μm cutoff filter, and then further clarified by centrifugation at 10,000 Xg to generate the APF. (C) Method #3 dissects leaves from the rosettes by cutting at the stem/petiole junction, vacuum infiltrating the leaves with APF extraction buffer, blotting dry the leaves, and collecting the APF by low-speed centrifugation (600 Xg) of the unbundled leaves with the petioles oriented up. The fluid was clarified with a 20-μm cutoff filter to generate the APF. For each method, the APFp could be isolated by high-speed centrifugation (100k Xg).


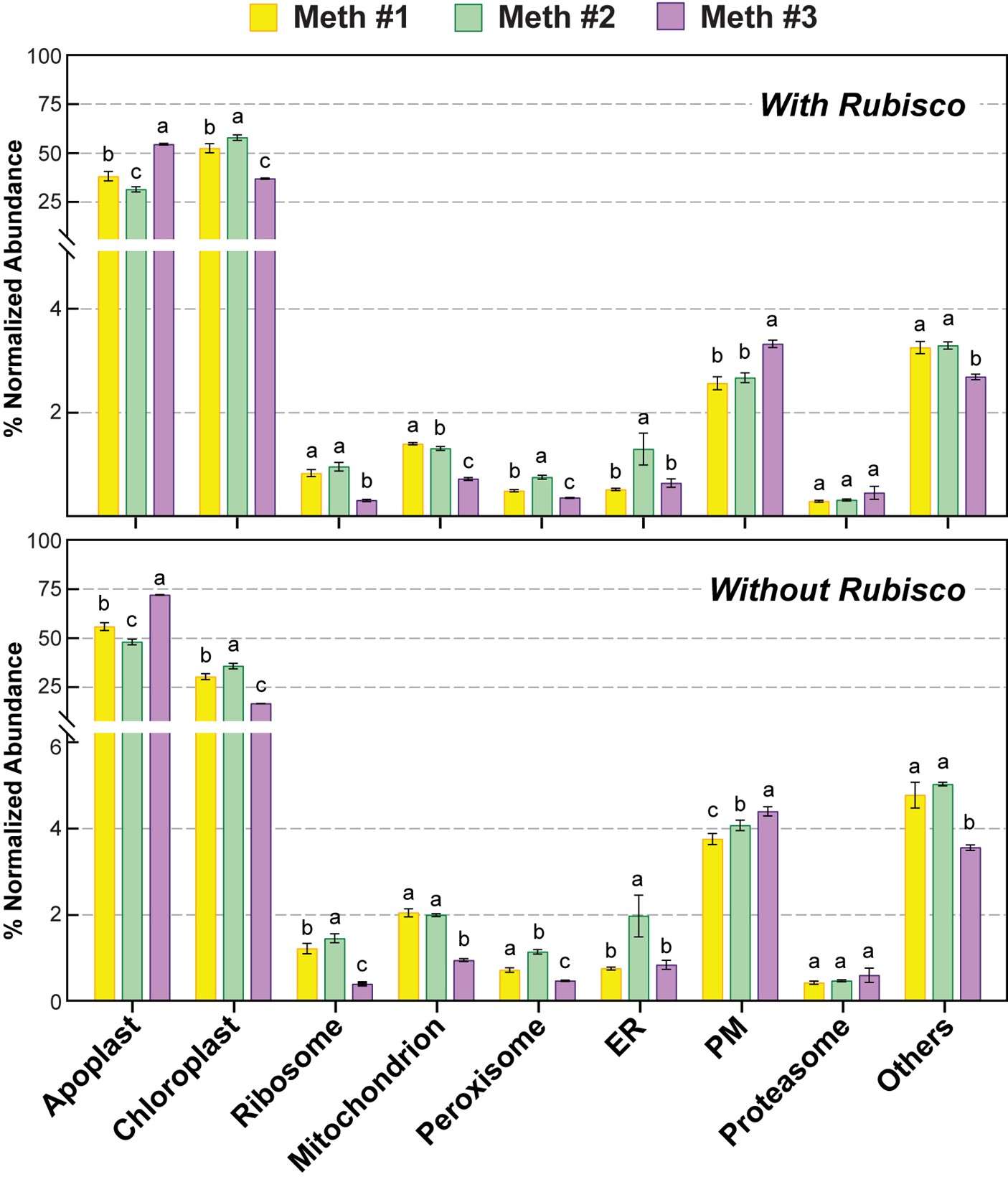


**Supplementary Figure S2 │** Comparisons of enrichment for Arabidopsis leaf proteins assigned to various cellular compartments/complexes following isolated by the three APF enrichment methods. Percent normalized abundances of proteins assigned to various cellular compartments/complexes as defined by GO and an apoplast protein catalog developed by Zand Karimi et al. (2025) were calculated from MS1 scans. The bar charts were created using the same data as Figure 2 with (top panel) or without (bottom panel) the values from Rubisco included. The values are means ± S.D. and different letters in each compartment/organelle represent significant differences (*p*-value < 0.05) as calculated by one-way ANOVA with Tukey’s post-hoc test.

**
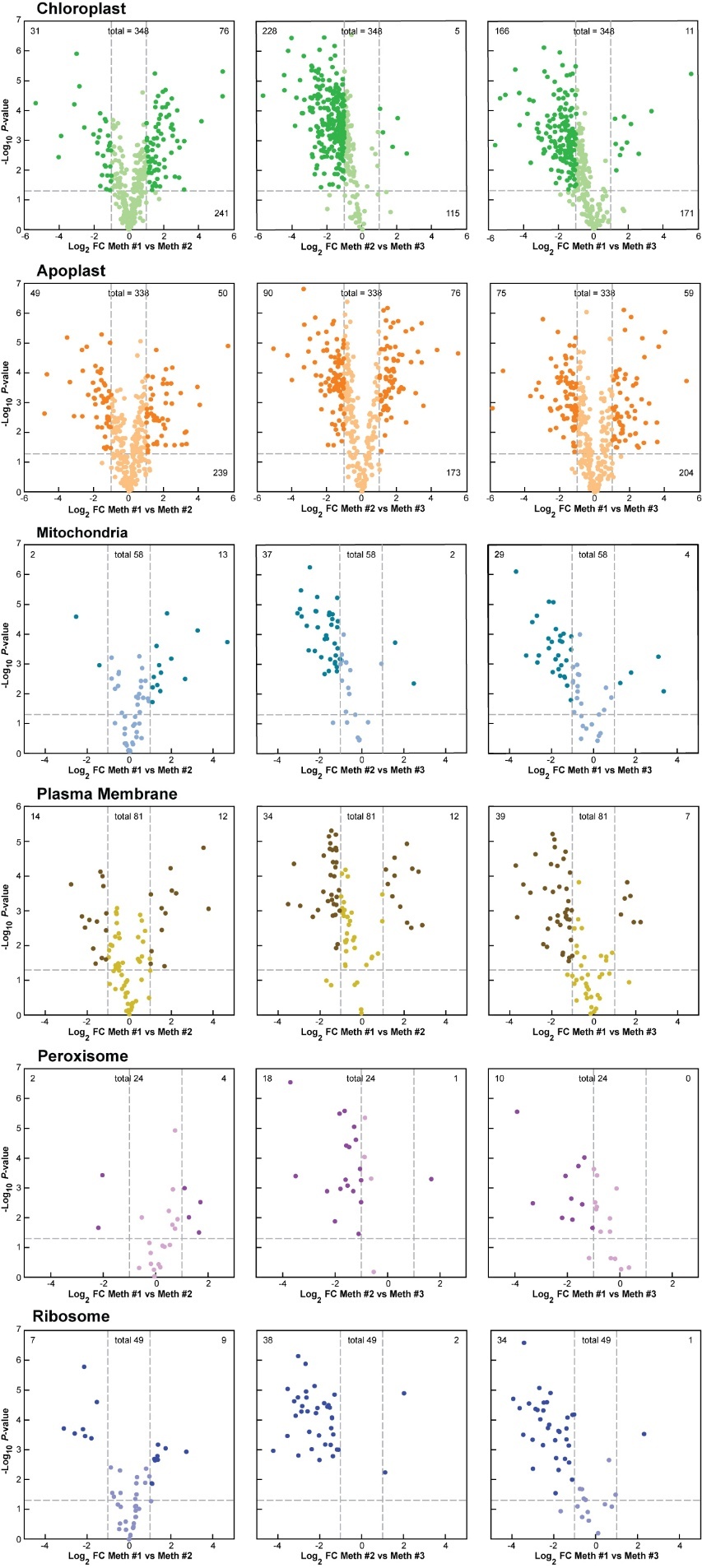
**

**Supplementary Figure S3│**Volcano plot comparisons showing the enrichment for various cellular compartments/complexes by the three APF isolation methods. Protein assignments were determined by GO in the APF isolated from Arabidopsis leaves either by recent methods (Rutter et al., 2017a) - Method 1 and (Huang et al., 2021) - Method 2), or by the improved method described here (Method 3). Three independent APF samples were prepared by each method and analyzed for protein composition by LC-MS/MS. Levels of proteins in common between samples were compared by Log_2_ FC in abundance in *p*-value of significance. The left panels compare Method 1 with Method 2, the middle panels compare Method 3 with Method 1, and the right panels compare Method 3 with Method 2. The darker colored dots indicate proteins with significant differences in levels between the two samples based on both Log_2_ FC ≧1 or ≦-1 and *p*-value of significance <0.05. The lighter-colored dots indicate proteins with insignificant differences in abundance between the two samples. The total numbers of proteins assigned to each compartment are indicated at the top. The numbers at the corners reflect proteins insignificantly or significantly more abundant in samples from one isolation method versus the other (Log_2_ FC ≧1 or ≦-1 and a *p*-value < 0.05).


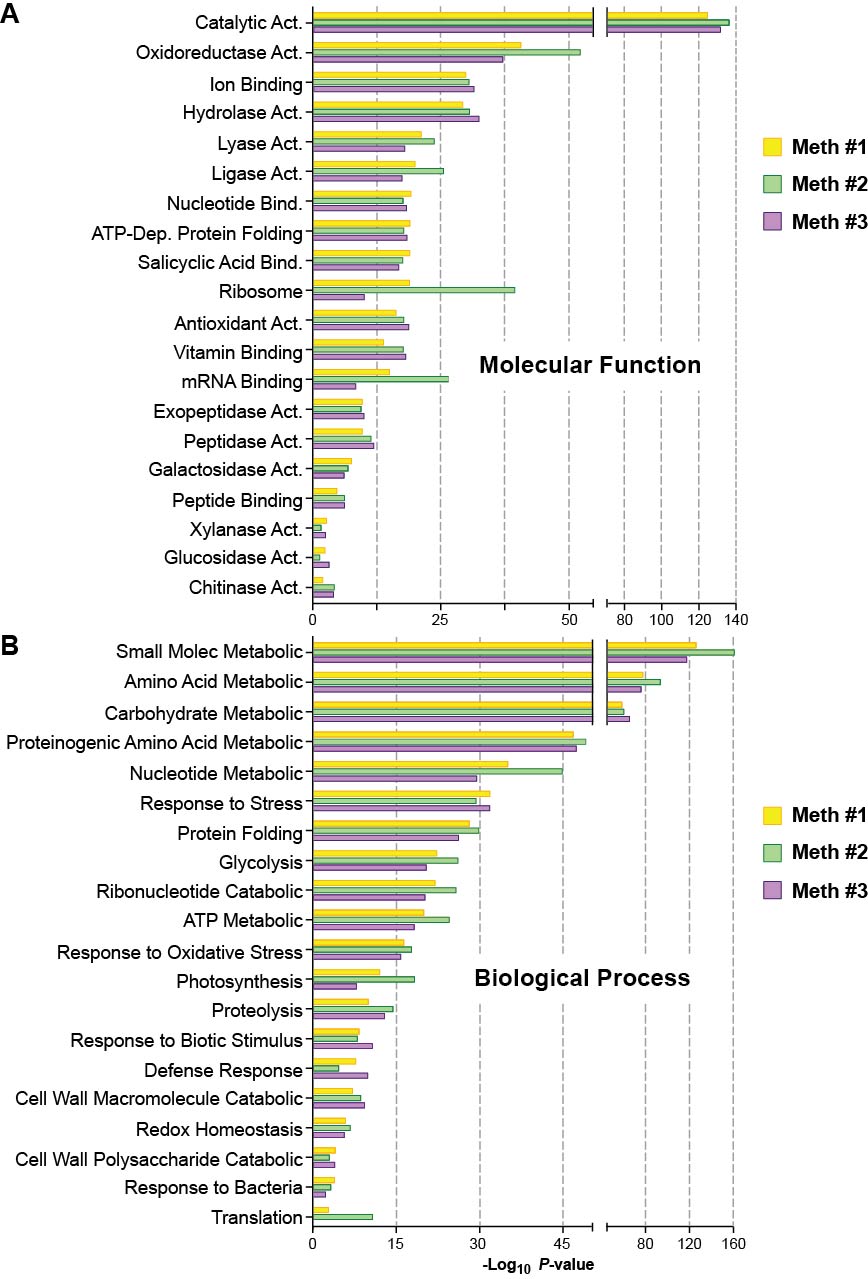


**Supplementary Figure S4 │**Gene Ontology (GO) enrichments based on the Molecular Function (A) and Biological Process (B) categories for the collections of proteins identified by LC-MS/MS in samples derived from the three APF isolation methods. GO enrichment was performed with the g:GOSt algorithm in the g:Profiler platform using the 1,471, 2022, and 1,551 proteins identified by Method 1, 2 and 3, respectively, after removing Rubisco polypeptides (Supplementary Datasets 2-4). The *P*-values of enrichment were calculated using multiple testing corrections and applications of the default g:GOSt algorithm to adjust for significance scores. Molecular Function and Biological Process terms were selected based on their uniqueness, statistical significance (*p*-value), and overall completeness of annotation.


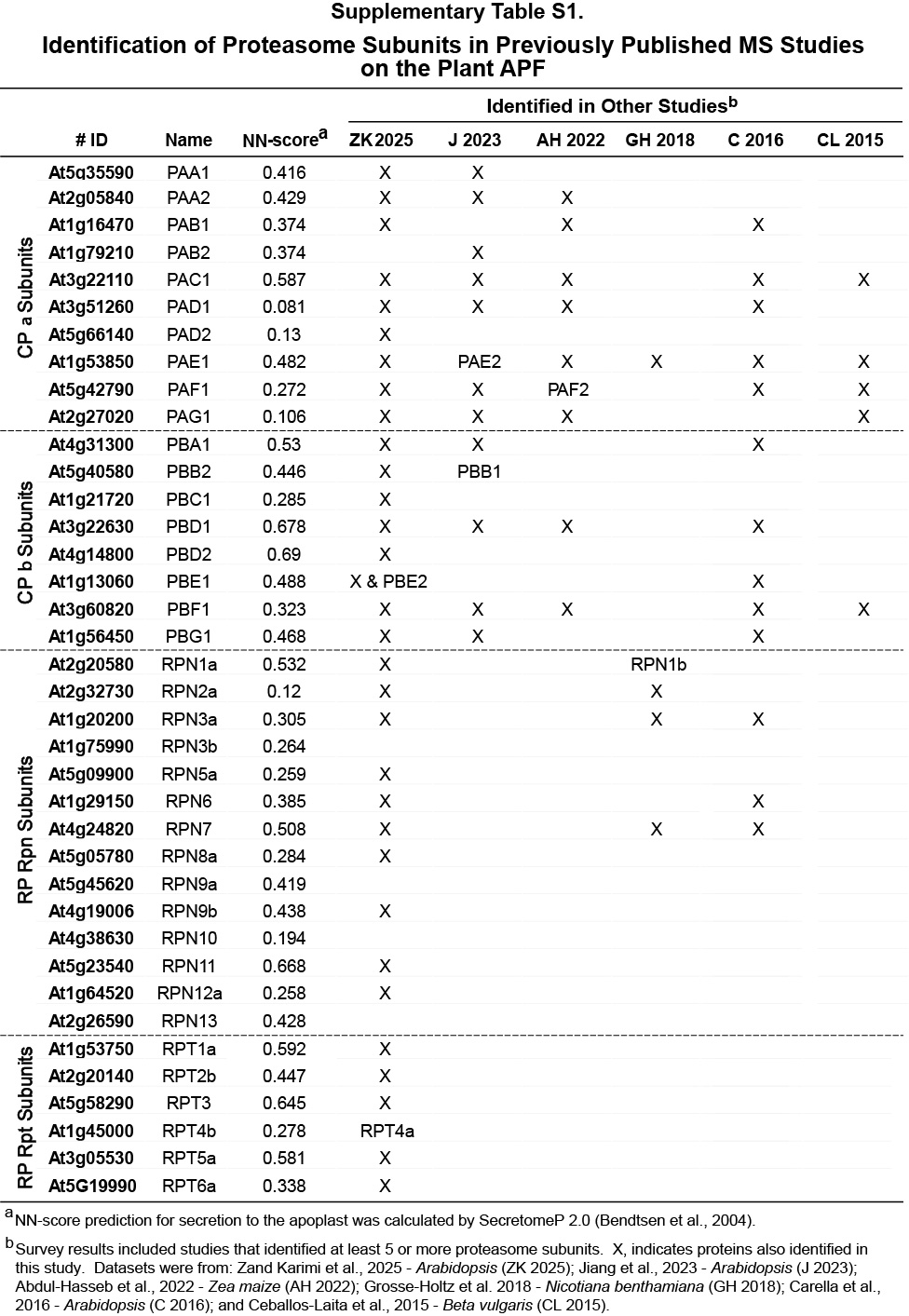

Supplement: Supplementary file 3 — Figure S1. Comparisons of the current VIC protocols for APF isolation from Arabidopsis leaves. Figure S2. Comparisons of enrichment for Arabidopsis leaf proteins assigned to various cellular compartments/complexes following isolated by the three APF enrichment methods. Figure S3. Volcano plots showing the enrichment for various cellular compartments/complexes by the three APF isolation methods. Figure S4. Gene Ontology (GO) enrichments based on the Molecular Function and Biological Process categories for the collection of proteins identified by LC–MS/MS in samples derived from the three APF isolation methods. Table S1. Identification of proteasome subunits in previously published MS studies on the plant APF. [file PLD3-9-e70087-s001.docx]
